# Mammal extinctions and the increasing isolation of humans on the tree of life

**DOI:** 10.1002/ece3.4630

**Published:** 2019-01-21

**Authors:** Sandrine Pavoine, Michael B. Bonsall, T. Jonathan Davies, Shelly Masi

**Affiliations:** ^1^ Centre d'Ecologie et des Sciences de la Conservation (CESCO) Muséum national d'Histoire naturelle (MNHN) Centre National de la Recherche Scientifique (CNRS) Sorbonne Université Paris France; ^2^ Mathematical Ecology Research Group Department of Zoology University of Oxford Oxford UK; ^3^ St Peter's College Oxford UK; ^4^ Department of Biology McGill University Montreal Quebec Canada; ^5^ African Centre for DNA Barcoding University of Johannesburg Johannesburg South Africa; ^6^ Departments of Botany, Forest & Conservation Sciences Biodiversity Research Centre University of British Columbia Vancouver British Columbia Canada; ^7^ Unité Eco‐anthropologie et Ethnobiologie (EAE) Muséum National d'Histoire Naturelle (MNHN) Centre National de la Recherche Scientifique (CNRS) Université Paris Diderot Paris France

**Keywords:** evolutionary history, extinction risks, phylogenetic originality, primates, threats

## Abstract

A sixth great mass extinction is ongoing due to the direct and indirect effects of human pressures. However, not all lineages are affected equally. From an anthropocentric perspective, it is often purported that humans hold a unique place on Earth. Here, we show that our current impacts on the natural world risk realizing that expectation. We simulated species loss on the mammalian phylogenetic tree, informed by species current extinction risks. We explored how *Homo sapiens* could become isolated in the tree if species currently threatened with extinction disappeared. We analyzed correlates of mammal extinctions risks that may drive this isolation pattern. We show that, within mammals, and more particularly within primates, extinction risks increase with the number of known threat types, and decrease with geographic range size. Extinctions increase with species body mass, trophic level, and the median longitudinal extent of each species range in mammals but not within primates. The risks of extinction are frequently high among *H. sapiens* close relatives. Pruning threatened primates, including apes (Hominidae, Hylobatidae), from the tree of life will lead to our species being among those with the fewest close relatives. If no action is taken, we will thus not only lose crucial biodiversity for the preservation of Earth ecosystems, but also a key living reference to what makes us human.

## 1 INTRODUCTION

Earth is experiencing a major extinction crisis (Barnosky et al., 2011). Marine and terrestrial ecosystems are undergoing profound changes as a result of human activities. Indeed, if primary causes for past mass extinction events still remain unclear, the anthropogenic cause of the current biodiversity crisis is widely understood (Novacek & Cleland, 2001). To evaluate the extent of this crisis, the loss of biodiversity is usually estimated in terms of number of species driven extinct. Notably, about one‐fifth of vertebrate species are threatened with extinction (Hoffmann et al., 2010). Yet, species are not equivalent, and not all lineages are affected equally.

For a conservation purpose, assigning species different conservation values might seem controversial. Giving all species, the same value equates biodiversity to the number of species (=species richness). However, we can simply reframe this problem by considering other hierarchies of biodiversity. For example, if we value each individual equally, we must value species differently as species are represented by different numbers of individuals (abundance‐based species diversity). Evaluating character states also naturally values species differently (character‐based diversity). An extension of this reasoning underlies Faith's (1992a,b) framework of phylogenetic diversity, where the “richness” of a species set is calculated by counting units of branch lengths in a phylogenetic tree, where branch lengths are assumed to capture changes in character states.

Phylogenetic diversity thus provides an alternative but equally robust measure of biodiversity that encapsulates the shared evolutionary histories of a set of species. The growing use of phylogenies in ecology and conservation biology recognizes that the branching pattern on a phylogenetic tree reflects the accumulation of some feature differences (e.g., morphological, behavioral, life history, and/or ecological differences) between evolutionary lineages (Harvey & Pagel, 1991; Tucker et al., 2017). These features are assumed (and sometimes proven to) display what is called a phylogenetic signal: Related species tend to resemble each other in their features, more than they resemble species drawn at random from a phylogenetic tree (e.g., Kamilar & Cooper, 2013). Thus, phylogenetic measures could be more effective than species‐based measures at preserving feature diversity (Faith, 1992a,b). Yet, current conservation practices may often poorly protect phylogenetic diversity as they target species‐rich areas with high endemism, but ignore species relatedness (Veron, Davies, Cadotte, Clergeau, & Pavoine, 2017).

The extinction risks of mammals have been particularly well studied compared to other groups, especially in a phylogenetic context (Veron et al., 2017). Including more than 5000 species, mammals are charismatic and essential key species for ecosystem functions. About a quarter of mammals are threatened with extinction (International Union for Conservation of Nature [IUCN] 2016). Losing even a single mammal species may represent the loss of millions of years of unique evolutionary history. For example, losing the near threatened Monito del Monte (*Dromiciops gliroides*, sole extant member of the Microbiotheria; IUCN, 2016) could represent a loss of about 63 MY of evolution (according to Fritz, Bininda‐Emonds, & Purvis, 2009 and Rolland, Condamine, Jiguet, & Morlon, 2014 phylogenetic estimations). However, future projections are even more dramatic: As extinction risks are clustered across the mammalian phylogenetic tree, entire lineages could disappear (Jono & Pavoine, 2012; Veron et al., 2017). For example, the loss of the threatened Sirenia (Dugong and Manatees; IUCN, 2016) would amount to a loss of 163 MY of evolutionary history.

Among the most endangered mammal lineages, it is particularly striking that more than half of our closest relatives, primate species, are currently under threat (Estrada et al., 2017). Here, we investigate how current extinction trends could modify the position of our own species, *Homo sapiens*, on the tree of life. We evaluate whether the observed nonrandom patterns of extinction risks could drive the future phylogenetic isolation (originality) of *H. sapiens*. A species is defined as original if it has few close relatives (Pavoine, Ollier, & Dufour, 2005). Currently, primates constitute one of the most species‐rich orders of mammals. However, among primates, the Hominidae family contains only seven species, all of them but *H. sapiens* are now threatened with extinction (IUCN, 2016). To understand better the future position of *H. sapiens* in the mammalian phylogeny, we explore a range of potential correlates of extinction risks: species’ phylogenetic distance to *H. sapiens*, their phylogenetic originality, *intrinsic* factors linked to species biology, and *extrinsic* factors linked to the threats that species face within their environment (e.g., urbanization, hunting) and across their species geographic distribution. In our analyses, we ask the following questions: How will extinctions impact the position of *H. sapiens* on the mammalian phylogenetic tree? Which factors correlate with species extinction risks?

## 2 METHODS

### 2.1 Mammalian extinction risk status

We used the IUCN Red List (IUCN 2016) to classify each mammal species into one of the following categories: least concern (LC), near threatened (NT), vulnerable (VU), endangered (EN), critically endangered (CR), data deficient (DD). We excluded extinct species and those extinct in the wild. We obtained classifications for 5451 mammal species of which 435 were primates. The IUCN Red List assessments rely on published data and expert inputs and may be subject to bias that might vary across taxonomic groups (Trull, Böhm, & Carr, 2018); however, the standardization of the data compiled enhances taxonomic comparisons (Rodrigues, Pilgrim, Lamoreux, Hoffmann, & Brooks, 2006).

### 2.2 Taxonomy and phylogeny

We used the Wilson and Reeder (2005) taxonomy following the IUCN Red List (IUCN 2016). For mammals, we used the Bininda‐Emonds et al. (2007) timetree improved by Fritz et al. (2009), referred to as the “Fritz et al. phylogeny” (4854 species had both a IUCN category and a position in this phylogeny). We also considered the phylogenetic tree published by Rolland et al. (2014) who resolved the Fritz et al.'s tree and redated it with dates from Meredith et al. (2011) (same species as in Fritz et al. phylogeny). We refer to the resulting timetree as the “Rolland et al. phylogeny.” For primates, we pruned the Fritz et al. and the Rolland et al. trees conserving primate species only (331 species). We also considered the four molecular phylogenetic trees from Springer et al. (2012) (340 primate species had both a IUCN category and a position in these trees). Springer et al. timetrees differed in their estimation of divergence times with the relaxed clock enforcing autocorrelated rates and hard‐bounded constraints (AUTOhard), autocorrelated rates and soft‐bounded constraints (AUTOsoft), independent rates and hard‐bounded constraints (IRhard), or independent rates and soft‐bounded constraints (IRsoft). We used different phylogenies to evaluate the robustness of our results to phylogenetic uncertainty. The phylogenetic trees we used differ in their methodology, in their underlying data, in their degree of resolution and in the number and identity of the included species.

### 2.3 Traits and geographic information

We characterized species by a number of factors that could explain, or at least correlate with, extinction risk. The first two factors concern potential phylogenetic signals in extinction risk: the phylogenetic distance of a given species to *H. sapiens* defined as the age of its first common ancestor with *H. sapiens*; and the phylogenetic originality of a species as measured by the evolutionary distinctiveness index (*ED*, Isaac, Turvey, Collen, Waterman, & Baillie, 2007), also known as the fair proportion index (Redding, Mazel, & Mooers, 2014).

Species affected by multiple threats could be more at risk (Jono & Pavoine, 2012). The IUCN Red List (IUCN, 2016) contains a classification of three levels of direct threats (Salafsky et al., 2008). Each first level is divided into several second‐level threats which are themselves subdivided into third‐level threats. The first and second levels “are designated to be comprehensive, consistent, and exclusive,” while the third level, by contrast, contains “illustrative examples rather than comprehensive listings of threats” (Salafsky et al., 2008). We considered here the number of ongoing second‐level threats affecting a species as these second‐level entries are the most detailed and complete.

Large body mass has been considered as a potential catalyst of species sensitivity (Cardillo, 2003; Fritz et al., 2009). Life‐history traits that increase species’ vulnerability to anthropogenic threats, such as lower population growth, long generation times, larger home ranges, low dispersal ability, frequently scale with body mass (Purvis, Gittleman, Cowlishaw, & Mace, 2000; Whitmee & Orme, 2013). We used the PanTHERIA database (Jones et al., 2009) complemented with the EltonTraits database (Wilman et al., 2014), which relies on Smith et al. (2003), to obtain body mass for 4479 species, among which 284 were primates. In the EltonTraits database, we excluded data identified as interpolated using genus or family averages to avoid any circularity in our analyses. This is an important exclusion if the taxonomy, which is likely to be, at least partially, correlated with the phylogeny, is used to predict traits to explain phylogenetic patterns in extinction risks.

The geographic range size of a species is one of the criteria used in the IUCN Red List to estimate extinction risk. We obtained geographic distributions from the IUCN Red List (IUCN, 2016). Each species’ known range is available as a polygon or a set of polygons in case of fragmented distributions. We selected only polygons where the species is currently known or thought very likely to occupy the area, which encompasses localities with current or recent (last 20–30 years) records with suitable remaining habitat at appropriate altitudes. We used the Mollweide projection to calculate the total area of these IUCN digital distribution maps for 5393 mammal species of which 421 were primates.

We obtained other traits and geographic information on species directly from PanTHERIA (Jones et al., 2009). In this database, we excluded variables with too many missing data (>60%) and redundant variables with absolute correlations higher than 0.65 (the phylogenetic distance to *H. sapiens* and the body mass having a correlation of 0.64 in primates). This selection process returned nine variables: diet breadth (number of dietary categories), habitat breadth (number of used habitat layers), litter size (number of offspring born per litter per female), trophic level (from herbivore [1], through omnivore [2], to carnivore [3]), median latitudinal extent of each species range, median longitudinal extent, mean human population density (persons per km²) in each species range, mean rate of increase in human population density, and mean monthly precipitation (mm). Diet and habitat breadth are related to the degree of specialization, a characteristic which has been associated with increasing extinction risks (Verde Arregoitia, Blomberg, & Fisher, 2013; and references therein). Small litters could be an indication for slow life histories. Organisms that occupy the highest trophic level are impacted by extinction risk of species lower down the food chain and they are more susceptible to the effects of pollution (Purvis, Gittleman, et al., 2000). Geographic variables (latitude, longitude) may reveal spatial differences in species’ extinction risk. Regarding climate variables, in a recent review, Verde Arregoitia (2015) found that precipitation showed significant association with extinction risk in almost half of the studies that included it. We did not consider temperature as an independent predictor because of its quadratic correlation with latitude. Finally, human density is an indicator of global anthropogenic impact often associated with mammal decline (Cardillo et al., 2004).

### 2.4 *Homo sapiens*’ current and future originality ranks

For each phylogenetic tree, we ordered species from the most to the least original (*ED* index). In case of ties, we attributed the average rank. We performed this ranking by first retaining all species (no extinction) and then with successive scenarios of species extinction. Under the most pessimistic scenarios, even species currently classified as near threatened may have a significant probability of extinction in the near future (e.g., Mooers, Faith, & Maddison, 2008), we thus simulated sequential extinctions focusing first on CR species only, next on EN and CR species, then on VU, EN, and CR species, and finally including NT, VU, EN, and CR species. We did not consider the extinction of LC species because this would lead to the simulated extinction of all mammal species. It has already been shown that extinction risks are not randomly distributed across the mammal phylogeny (Cardillo, Mace, Gittleman, & Purvis, 2006; Jono & Pavoine, 2012; Purvis, Agapow, Gittleman, & Mace, 2000; Russell, Brooks, McKinney, & Anderson, 1998; Veron et al., 2017). We here evaluated the hypothesis that the originality of *H. sapiens* under each scenario of species extinction is higher than the originality expected if the extinction risks of mammals were random among the tips of the phylogenetic tree. For each scenario of species extinctions, we used a Monte Carlo approach to test this hypothesis, with the following steps: step#1. we simulated species extinctions according to the selected scenario of extinction, and we calculated the originality rank for *H. sapiens* among the surviving species (*Obs*.); step#2. we randomly permuted the extinction risk status among all nonhuman mammals, simulated species extinctions according to the same selected scenario and calculated the originality rank for *H. sapiens* (*Sim*.) among the surviving species; step#3. we repeated step#2 500 times; step#4. we calculated the *p*‐value as the proportion of time *Sim*. was lower than or equal to *Obs*. As a first approach, we excluded, from our analyses, species with missing phylogenetic positions. For data‐deficient species in the IUCN Red List, we considered extreme scenarios where these species were either all LC or all CR. We performed the same analyses but on primates only and 1000 permutations at step#3. We also repeated these tests with Lapointe and Garland (2001)'s phylogenetically controlled permutations of extinction risks. The Lapointe and Garland algorithm permutes extinction risk status between species according to the amount of branch length separating them in the phylogeny: Two closely related species are more likely to exchange status than two more distantly related species. The algorithm depends on a parameter, *k*, that determines how much the permutations of extinction risk status depend on the amount of branch length separating two species in the phylogeny. *k* ranges from 1 (permutations are strongly constrained by the phylogeny) to ∞ (equally likely permutations). We use here *k *=* *1 (the other extreme case, *k * = ∞, corresponding to our first run of the Monte Carlo approach, where permutations are not constrained by the phylogeny).

As additional sensitivity analyses, we randomly assigned phylogenetic positions to all phylogenetically unplaced species and imputed extinction risk status (via the missForest algorithm, see details below) to species classified as data deficient in the IUCN Red List. This was done to test the robustness of our results to missing data. In the phylogenetic trees, we randomly connected missing species to the smallest monophyletic clade (subtree) that contained all available species from their family. The probability that a new tip (here species) is added along any branch was directly proportional to the length of the branch. The order in which species were added was randomized. We repeated this operation 200 times for mammals and 500 times for primates. As we analyzed two phylogenetic trees for mammals and six for primates, we ended up with the simulation of 200*2 = 400 complete trees for mammals, and 500*6 = 3000 complete trees for primates.

To impute extinction risk status for data‐deficient species, we collected, for each other species the extinction risk status and for all species, including data‐deficient species, variables already known to be strongly linked with probabilities of extinction and for which many data are available: taxonomy (order when we considered all mammals, family when we focused on primates), body mass, and geographic range size (Cardillo, 2003; Cardillo et al., 2005; Purvis, Agapow, et al., 2000). Then, we used the missForest algorithm to impute missing extinction risk values (Stekhoven, 2013; Stekhoven & Buehlmann, 2012). The missForest algorithm was repeated 200 times for mammals and 500 times for primates to account for uncertainties in predictions.

We thus generated 400 data sets (200 repetitions of the randomly completed trees, each associated with a completed vector of extinction risks for each core phylogenetic tree [the Fritz et al. and the Rolland et al. phylogenies]) with a phylogeny and a vector of extinction risk status for 5451 mammal species, and 3000 data sets with a phylogenetic tree and a vector of extinction risk status for 435 primate species. These data sets are not estimations of true phylogenies and status but are tools to evaluate the robustness of our results to uncertainties due to missing data.

We applied the analysis above on *H. sapiens*’ originality to each data set reducing the number of permutations in Monte Carlo tests to 200 for mammals and 500 for primates given the high number of replicate data sets treated and the large size of the mammal phylogeny. This led to the calculation of 320,000 vectors of originality for mammal species (400 data sets * 4 extinction scenarios * 200 permutations per scenario) and 6,000,000 vectors for primate species (3000 data sets * 4 extinction scenarios * 500 permutations per scenario).

We performed all analyses in R (R Core Team, 2017), using a nominal α level of 0.05 for all our tests.

### 2.5 Multivariate model of species’ extinction risks

We developed phylogenetic models in a Bayesian MCMC framework (Hadfield, 2010) where the IUCN extinction risk status was the response expressed as an ordinal variable from 0 (least concern) to 4 (critically endangered). We used this ordinal classification for the response trait (=extinction risk) distribution and the Gelman prior for ordinal regression (Gelman, Jakulin, Pittau, & Su, 2008) implemented in R (Hadfield, 2010). The explanatory variables were all factors listed in Section “Traits and geographic information.” For the degree of phylogenetic originality of each species, we calculated the median species originality over the 200 (for mammals) or 500 (for primates) simulated data sets with randomly attributed phylogenetic positions and imputed IUCN status to all species with missing data (see Section “*H. sapiens*’ current and future originality ranks”). Only species with known phylogenetic positions and IUCN status are considered in the MCMCglmm approach. However, these simulations allowed us to account for potential effects of missing data on the relative originalities of each of these species. We treated the median latitudinal and longitudinal extent of each species range by orthogonal polynomials of degree 1 and degree 2 and log‐transformed body mass and species originality. We scaled all variables to a mean of 0 and a variance of 1. We acknowledged that geographic range size is one of the criteria used by the IUCN Red List to define categories of extinction risks. As in Cardillo et al. (2005), we thus also ran our models after having removed species not listed under criterion A of the Red List (a measurable decline in population size). In addition, we identified that the classification into herbivory and omnivory seemed indistinct for some primate species in the PanTHERIA database. We thus also repeated the MCMC approach without the two explanatory variables, diet breath and trophic level.

## 3 RESULTS

Our results were robust with respect to missing species and phylogenetic uncertainty, as we obtained similar main conclusions with all tested phylogenies and with different treatments of missing extinction risk status. We summarize results here for the Rolland et al. phylogeny for mammals and the Springer et al. AUTOhard phylogeny for primates, results across all phylogenies are presented in the Supporting Information Appendix S1.

### 3.1 *Homo sapiens* is likely to become a phylogenetically isolated species

**Figure 1:**
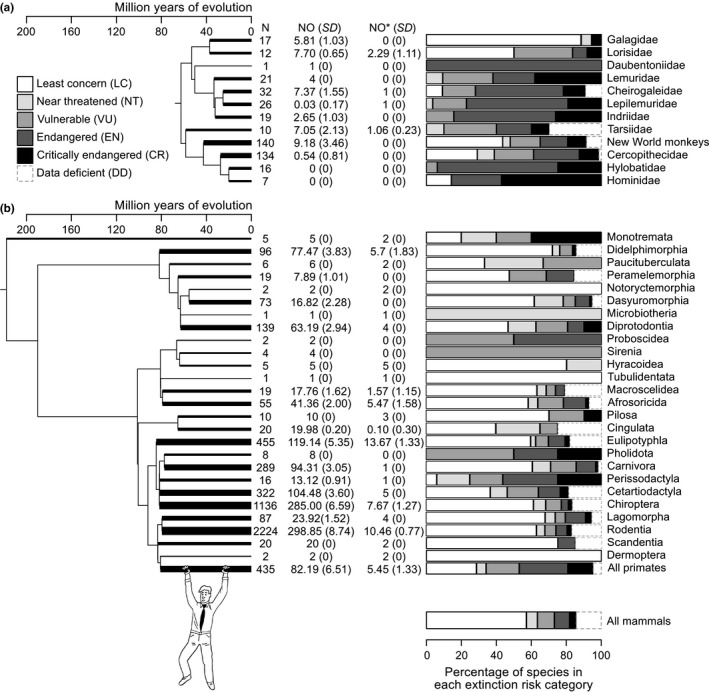
Illustration of phylogenetic patterns in extinction risks and in the number of species that are more original than *Homo sapiens* in (a) primates and (b) mammals. We used the Springer et al. tree in (a) and the Rolland et al. tree in (b) to provide partial representations of the primate and mammal phylogeny. In (a), tips of the tree are the primate families except for the paraphyletic New World families (Atelidae, Aotidae, Callitrichidae, Cebidae, Pitheciidae) that we grouped into a single clade. In (b), tips of the tree are the monophyletic mammal orders. Given that we displayed a simplified version of the trees, we used the thickness of the terminal branches in trees to better indicate how many species there are in each family (for primates) or order (for mammals). The thickness is equal to log(1 + *N*)/log(2)*u, where *N* is the number of species, and *u* is the basic thickness when *N* = 1. Next to each phylogenetic tree, a table gives the number of species in each terminal clade (*N*) including data‐deficient species (IUCN 2016), the number of species (NO) that were more original than *H. sapiens* according to index ED in our simulations of missing data effects and its standard deviation over all simulations (*SD*), the same number of species (NO*) if currently threatened species are driven extinct (vulnerable, endangered and critically endangered species). A bar plot gives the percentage of species in each IUCN category (IUCN 2016)

We estimated the current originality rank for *H. sapiens* to be 1332 (i.e., humans are the 1332nd most original species) among 5451 mammal species and the 46th most original species among 435 primate species. If CR species were the only set of mammals to disappear, the relative originality of *H. sapiens* would increase (Table 1). If both CR and EN species were driven extinct, *H. sapiens* would rank among the 10 most original primate species, a rank that would be maintained if VU and NT species were also driven extinct (Table 1; Figure 1). If CR and EN species were driven extinct, *H. sapiens* would rank among the 200 most original mammal species. If all threatened (VU, EN, and CR) species were lost, *H. sapiens* would rank among the 100 most original mammal species, a rank that would be maintained if NT species were also driven extinct (Table 1; Figure 1). Such a dramatic alteration in rank would be highly unlikely if the threatened species were randomly distributed across the phylogeny (Table 1), even when accounting for phylogenetic autocorrelation (Supporting Information Appendix S1).

**Table 1:** Estimates of *Homo sapiens* originality rank if currently threatened and near‐threatened species were driven extinct and comparison with random extinctions

Group	Extinctions	Nb. of species	Originality rank^a^	
			*Obs*.^b^	Mean *Sim*. (*SD*)
Mammals	None	5451	1332	
	CR	5138	991**^c^	1282 (67.54)
	EN, CR	4456	158***	1161 (123.67)
	VU to CR	3785	81***	1024 (154.77)
	NT to CR	3373	82***	931 (169.10)
Primates	None	435	46	
	CR	369	32*	42 (5.02)
	EN, CR	244	5***	33 (8.08)
	VU to CR	157	6*	24 (8.57)
	NT to CR	131	4**	22 (8.37)

^a^Results here are averaged over all our simulations, which account for missing data (detailed results in Supporting Information Appendix S1). ^b^We first measured *Obs*., the observed rank for the originality of *H. sapiens*, ordering species from the highest to the lowest originality. Next, we drove all critically endangered (CR) species to extinction and performed the same calculation. We compared the observed rank for *H. sapiens* originality with ranks obtained permuting extinction risks between mammals (200 times) or primates (500 times). The average simulated rank (*Sim*.) for *H. sapiens* and its standard deviation (*SD*) are given. We repeated this approach driving endangered (EN) and CR species, next vulnerable (VU) to CR, and then near threatened (NT) to CR species to extinction. ^c^Proportion of times *Sim*. was lower than or equal to *Obs*.: *0.01 < *p* ≤ 0.05, **0.005 < *p* ≤ 0.01, ***p ≤ 0.005.

**Table 2:** Ordinal phylogenetic model in a Bayesian MCMC framework testing the potential effects of 16 specified explanatory variables on species extinction risks

Group	Variable^a^	Posterior mean	CI	pMCMC
Mammals	Distance to *Homo sapiens* b	−0.283	[−1.282; 0.679]	0.504
	Originality	0.095	[−0.081; 0.315]	0.400
	Nb of known threats^c^	1.104	[0.927; 1.299]	<0.004
	Body mass	0.945	[0.546; 1.256]	<0.004
	Geographic range size	−1.301	[−1.616; −1.050]	<0.004
	Diet breadth	−0.023	[−0.185; 0.161]	0.792
	Habitat breadth	0.070	[−0.090; 0.270]	0.424
	Litter size	−0.082	[−0.296; 0.157]	0.496
	Trophic level	0.305	[0.059; 0.534]	0.016
	Med. latitude (deg. 1)^d^	0.124	[−0.098; 0.324]	0.248
	Med. latitude (deg. 2)^d^	0.086	[−0.118; 0.298]	0.440
	Med. longitude (deg. 1)^d^	0.234	[0.035; 0.460]	0.016
	Med. longitude (deg. 2)^d^	−0.083	[−0.296; 0.121]	0.456
	Human pop. density^e^	0.087	[−0.020; 0.215]	0.144
	Human pop. change^f^	0.067	[−0.094; 0.192]	0.368
	Precipitation	0.032	[−0.136; 0.209]	0.688
Primates	Distance to *H. sapiens* b	−0.059	[−3.445; 3.426]	0.969
	Originality	1.404	[−0.463; 3.696]	0.131
	Nb of known threats^c^	2.883	[0.490; 5.651]	<0.001
	Body mass	1.503	[−1.312; 4.328]	0.245
	Geographic range size	−3.880	[−6.330; −1.505]	≪0.001
	Diet breadth	−0.598	[−2.441; 1.033]	0.456
	Habitat breadth	0.764	[−0.669; 2.532]	0.284
	Litter size	−0.162	[−2.721; 1.968]	0.973
	Trophic level	−0.461	[−2.459; 1.338]	0.573
	Med. latitude (deg. 1)^d^	0.629	[−1.342; 2.898]	0.539
	Med. latitude (deg. 2)^d^	−0.723	[−2.439; 0.883]	0.332
	Med. longitude (deg. 1)^d^	−0.206	[−3.274; 2.566]	0.920
	Med. longitude (deg. 2)^d^	−0.786	[−3.177; 1.213]	0.476
	Human pop. density^e^	1.296	[−0.272; 3.168]	0.096
	Human pop. change^f^	−0.328	[−2.187; 1.324]	0.714
	Precipitation	−0.460	[−1.955; 0.916]	0.536

^a^See the Methods section and the database PanTHERIA, Jones et al. (2009), for details on each variable (all variables were scaled to a mean of 0 and a variance of 1); ^b^In Million years of evolution; ^c^Number of known threat types affecting a species; ^d^Median (Med.) latitudinal extent of each species range and median longitudinal extent of each species range, each expressed as orthogonal polynomials of degree (deg.) 1 and 2; ^e^Human population density; ^f^Mean rate of increase in human population density.

All mammal orders have at least one species that is currently more original than *H. sapiens* (Figure 1). When we simulated the loss of threatened species, the number of these species drastically decreased. In this scenario, we predict that only a few surviving primates will be more original than *H. sapiens* (Figure 1): the western fat‐tailed dwarf lemur (*Cheirogaleus medius*), the weasel sportive lemur (*Lepilemur mustelinus*), and the Philippine tarsier (*Tarsius syrichta*) (in 100% of our simuations). Other species were more original than *H. sapiens* in some fraction of our simulations: the pygmy tarsier (*Tarsius pumilus*, in 6% of the simulations); the east, Milne‐Edwards's and west African pottos (*Perodicticus ibeanus*, 40%; *P. edwardsi*, 43%; *P. potto*, 71%); and the gray slender loris (*Loris lydekkerianus*, 71%). *Tarsius pumilus* is a data‐deficient species in the IUCN Red List; and this species, together with *P. ibeanus* and *P. edwardsi*, had unknown phylogenetic position. The results for these species are directly dependent on our robust treatment of missing data.

### 3.2 Intrinsic and extrinsic factors that correlate with species extinction risks

Data on phylogeny, and all biological and geographic variables were available for 1251 mammals (about 23% of all species listed in the IUCN Red List), among which 92 were primates (about 21% of all primates listed in the IUCN Red List). Within mammals, the number of threats, body mass, and trophic level (from herbivore, through omnivore, to carnivore) and the median longitudinal extent of each species range were positively correlated with the IUCN extinction risk status (from least concern to critically endangered; Table 2). As expected, extinction risk decreased with geographic range size (Table 2). Within primates, only the number of threat types was additionally correlated with extinction risk (Table 2). We also observed that extinction risks in primates tended to increase with the degree of phylogenetic originality (pMCMC < 0.20; Table 2 and Supporting Information Appendix S1). However, this trend was significant only with one of our phylogenies, the pruned Rolland et al. tree, and only when considering extinction risks linked to an observed reduction in population size (criterion A of the Red List) (pMCMC<0.05; Supporting Information Appendix S1). Considering only extinction risks linked to an observed reduction in population size did not alter our main findings; however, primate extinction risks also significantly increased with human population density and the influence of trophic level and the median longitudinal extent of each mammal species on extinction risk levels were only marginally significant (pMCMC<0.10; see Supporting Information Appendix S1). The removal of diet breath and trophic level from the explanatory variables impacted our results only when we excluded species not listed under criterion A of the Red List: Habitat breadth and precipitation became significant when considering all mammals; and primate extinction risk significantly increased with species originality with the pruned Rolland et al. tree only (Supporting Information Appendix S1).

## 4 DISCUSSION

Under a scenario of future extinctions, we demonstrated that *H. sapiens* will become a phylogenetically original species among mammals and also within primates. We found no evidence that extinction risk increases with the phylogenetic proximity to *H. sapiens*. However, significant predictors that do correlate with mammalian extinction risk (and are correlated directly or indirectly with human actions) include small geographic range size and high number of threat types in mammals and within primates, high body mass, high trophic level (from herbivores to carnivores) in mammals but not within primates alone, and sharing the habitat with high human population density in primates (Cardillo et al., 2004; Fritz et al., 2009; IUCN, 2016; Jono & Pavoine, 2012; Purvis, Gittleman, et al., 2000). The observed increased extinction risk for mammals with longitude (from America, through Europe and Africa, to Asia and Oceania) was less expected. We did not observe this trend within primates. Most likely, longitude represents a proxy variable for other spatially structured predictors. There are several possible reasons that could explain why we did not detect a similar relationship for primates. First, primates may be more sensitive to multiple threats compared to other mammals, thus some differences between primates and other mammal orders are not surprising. Second, primates are mostly tropical, and perhaps the longitudinal influence is stronger outside of the tropics. Third, primates are neatly split phylogenetically with New World species in the west and Old World species in the east. Among the most endangered species, apes only occur in Africa and Asia, and lemurs in Madagascar. This geographic separation of major clades might mask any longitudinal gradient in extinction risk.

While some of the most original mammal species are threatened with extinction (e.g., the aye‐aye [*Daubentonia madagascariensis*], Sir David's long‐beaked echidna [*Zaglossus attenboroughi*], eastern long‐beaked echidna [*Z. bartoni*], western long‐beaked echidna [*Z. bruijnii*]), a trend for increased extinction risk among more original species was significant in only one of the phylogenies we considered (the pruned Rolland et al. tree) and only when considering primates alone. Verde Arregoitia et al. (2013) found an increase in extinction risk for evolutionarily distinctive primates; however, our results show that a high gain in originality for a species is not necessarily accompanied by high correlations between extinction risk and species originality. This difference between the Verde Arregoitia et al. (2013) study and ours is likely due to the crucial improvement in the knowledge of primate species extinction in 2014, 2015, and 2016: Among the 435 primate species analyzed in our study, the IUCN status of 272 species was assessed in 2008, four species in 2011, two species in 2012, a single species in 2013, 101 in 2014, 20 in 2015, and 35 in 2016 (IUCN, 2016). In addition, we did not find a direct link between extinction risk and phylogenetic proximity to *H. sapiens* (see Appendix 1). The future originality of our species is thus likely a consequence of both the extinction of some, but not all, of the currently original species, and the endangerment of our closest species, while nonprimate extinction risks are sufficiently scattered across the mammalian phylogeny such that only a few other species will be more original than humans in the tree of surviving (nonthreatened) species. Future predictions could be even more severe if current trends continue as three of the surviving primate species that we predict to be more original than *H. sapiens* (*C. medius, L. mustelinus, and T. syrichta*) are endemic species from Madagascar with a continuing decline in extent of occurrence and/or in population size (IUCN, 2016). Two of them (*L. mustelinus and T. syrichta*) are already classified as near threatened (IUCN, 2016). Furthermore, all those species are prosimians, thus belonging to the most phylogenetically distant primate taxon from *H. sapiens*. This further underlines the future isolation of *H. sapiens* in the phylogenetic tree.

Given the high number of species at risk of extinction and limited conservation resources, we cannot avoid the “agony of choice” and the opportunity costs of selecting which species or habitat to protect (Vane‐Wright, Humphries, & Williams, 1991). Among the many potential criteria used to prioritize conservation actions, the originality of a species is increasingly recognized as important as phylogenetically original species may represent rare biological features that few other species possess (Isaac et al., 2007). It is ironic, therefore, that *H. sapiens* is likely to be a highly original species among living mammals in the near future, and one of the most original primate species. It is all the more ironic that our study shows we, humans, might be the driver of our own phylogenetic distinctiveness by endangering the existence and heightening the extinction risk of our closest mammalian relatives. Original species have often been likened to living fossils or taxonomic relicts. However, humans are neither living fossils nor relicts. Our study shows that high species originality can be a product of recent extinction, and thus, the originality of a species is independent of its age (Grandcolas, Nattier, & Trewick, 2014). By pruning the tree of life around us, we are increasing our originality and thus increasing the rarity of our biological characteristics, as well as losing evolutionary insights into their origins.

The pruning of the primate tree started well before the emergence of *H. sapiens*. Apes were much more diverse in the Miocene than now. By the end of the Miocene, the Earth had become littered with extinct ape lineages (Lovejoy, 2007). The same trend is seen in hominins: *H. sapiens* is the sole member of a much larger evolutionary bush of hominin species (Haile‐Selassie, Melillo, & Su, 2016). Why other hominins went extinct is still controversial. In the case of *Homo*, one of the existing hypotheses is that *H. sapiens* was the cause of the extinction of other close relatives, such as Neanderthals (*H. neanderthalensis)* (Churchill, Franciscus, McKean‐Peraza, Daniel, & Warren, 2009). Perhaps these extinctions were an early forerunner of what is to come.

A recent study suggests that extinction rates throughout primate history decline over time (Arbour & Santana, 2017). However, we suggest that the rhythm of primate extinctions is likely to change dramatically: by converting IUCN categories into probabilities that a species go extinct in the next 50, 100, or 200 years (Mooers et al., 2008), our study shows that extant primates have high risk of rapid extinctions, with our closest relatives being among the most likely to be lost first. It is not immediately obvious, however, why we should care that we are becoming increasingly phylogenetically original. We suggest that our close living relatives shed light on the origins of our diseases, our adaptations, especially regarding our unique cognitive capacities, our behaviors (the most brilliant and the most peculiar ones, including spite and war, Wilson et al., 2014), our capacity of self‐medication, to have culture, to communicate with complex language, and much more that we cannot discern from fossils. Their loss is our loss.

Due to their high risk of extinction, designing appropriate conservation strategies for our close relatives is critically important, but also requires consideration of the many costs to local people (Hill, 2002). While primates act as important seed dispersers to regenerate and maintain the forest ecosystem like other large frugivorous mammals (Chapman & Russo, 2006), they can also act as disease reservoirs (Woodford, Butynski, & Karesh, 2002) and as pests raiding crops (Tweheyo, Hill, & Obua, 2005) and even raiding our homes (Fuentes & Wolfe, 2002). The interactions between humans and nonhuman primates are numerous. As human cultures, needs and priorities constantly change, so do these interactions, challenging conservation actions that must constantly adapt to reflect these shifts.

Through our actions, we are isolating ourselves. Our study shows the future evolutionary trajectory of our loneliness. With the loss of our close relatives, we lose not only unique biodiversity that is essential to maintain ecosystem functions and the Earth climate but also a sense of our own fragility, our connection with the environment, reinforcing our delusions of success. Our actions blur the question of what makes us distinct, what makes us “humans.” Extinctions will leave us without a mirror to contextualize our biology. They accentuate the misconception that we are unique and then, ironically lead us to fulfill it.

## CONFLICT OF INTEREST

No competing interests to declare.

## AUTHOR CONTRIBUTIONS

SP designed the study, performed the analyses, and wrote the first version of the study. All authors commented and discussed on the results and contributed critically to the final version of the study.

## Figures and Tables

**Figure 2:**
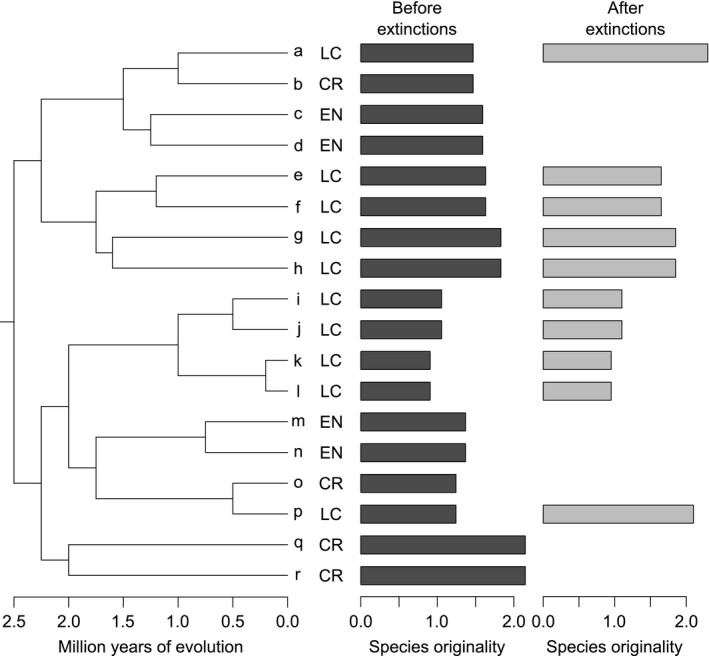
Illustration of the effect species extinctions may have on species originality thanks to a theoretical example. Here we considered a set of 18 species named from letter *a* to letter *r* and their theoretical phylogenetic tree. We provide the extinction risk status of each species next to its name (LC: least concern; EN: endangered; CR: critically endangered). Two bar plots in front of the phylogeny indicate the level of originality of each species according to index ED, first (dark gray bars) with all species and second (light gray bars) when currently threatened species (classified CR and EN) are dropped out from the phylogenetic tree

## Data Availability

For our study, we used data from the International Union for Conservation of Nature Red List, and from electronic appendixes of five papers, as specified in the Methods section. Upon acceptance, we will deposit all data and all R scripts in Dryad Digital Repository so that results are fully reproducible.

## APPENDIX 1 Theoretical example of the effect species extinctions may have on the level of originality of surviving species

### 1.1

In the theoretical example of Figure A1, at the beginning, species *q* and *r* are the most phylogenetically original species. However, the extinction of currently threatened species (classified as CR and EN) leads to the extinction of these two species together with six other species (named *b*,* c*,* d*,* m*,* n*, and *o*). Among the remaining species, species *a* becomes the most original followed by *p*. At the beginning (without extinctions), they were the 9th (ex equally with *b*) and the 13th (equally with *o*) most original species, respectively. It can be observed that the new rank of originality of species *a* is only partially due to an association between extinction risk and phylogenetic proximity to this species. Indeed, other species close to species *p* also were highly threatened together with the two very original species named *q* and *r*.

As a conclusion, the loss of close species and the loss of original species in the phylogeny may increase the observed originality of a species compared to other surviving species. A currently nonoriginal species could become the most original in the future if other currently original species disappear, if its closest species are lost, and if all other extinction risks are sufficiently scattered in the phylogeny so that no other species become more original. The theoretical example in Figure A1 thus shows that a high gain in originality for a given species is not necessarily accompanied by a high correlation between extinction risk and phylogenetic proximity to that focal species.
