# Supplementary material for: Mammal extinctions and the increasing isolation of humans on the tree of life
Source: Ecol Evol. 2019 Jan 21;9(3):914–24. doi: 10.1002/ece3.4630 (PMC6374672; doi:10.1002/ece3.4630)
Supplement: Supplementary file 1 [file ECE3-9-914-s001.docx]

"Mammal extinctions and the increasing isolation of humans on the tree of life" by Sandrine Pavoine, Michael B. Bonsall, T. Jonathan Davies, and Shelly Masi

**Appendix S1**

**Table S1-1.** *Homo sapiens* phylogenetic originality among mammals under different scenarios of species extinctions, accounting for missing data. Extinction risk status for species were LC=least concern, NT=near threatened, VU=vulnerable, EN=endangered and CR=critically endangered. Extinction risks for data deficient species were imputed thanks to the missforest algorithm and information on species orders, geographical and biological characteristics. Species with missing phylogenetic positions were randomly attached to their family sub-tree or to the sub-tree of the smallest monophyletic group that contains all species from their family. We simulated 200 data sets with phylogenetic positions for all species available in the IUCN Red List and with extinction risk status also for all species. Then for each data set, we considered the *ED* index of phylogenetic originality. Obs. is the observed rank for the originality of *H. sapiens* (where species are ordered from the highest to the lowest originality). Next we drove all CR species to extinction, and performed the same calculations. We repeated this approach when EN and CR species, next VU to CR and then NT to CR species are driven extinct. We compared Obs. with the rank for the originality of *H. sapiens* obtained when permuting (200 times) the IUCN status between all mammal species, except *H. sapiens* which conserved the LC status. Observed and simulated ranks had bell-shaped distributions. For each phylogeny, each scenario of species loss, and each originality index, we provide the mean of Obs. and its standard deviation (SD) over the 200 simulated data sets, the mean of the simulated values (Sim.) and its standard deviation over the 200 simulated data sets. Each data set led to the calculation of one Obs. and 200 Sim. values, the standard deviation of Sim. calculated for each data set is also important as Obs. is compared to the 200 Sim. values per data set. To better describe our results in details, we thus also calculated, for each data set, the standard deviation of Sim. values, which led to 200 standard deviations for which we calculated the mean (MSD) and own standard deviation (SSD). The number of species remaining after each scenario of species loss varied between data sets to account for uncertainties when imputing extinction risk status to data deficient species; this number of species had a bell-shaped distribution, we thus provide its mean and standard deviation over the 200 simulated data sets. In the main text, we chose to present only averaged values over data sets: mean of Obs., mean of Sim. and of its standard deviation (MSD) and mean of the number of species. The P-value was calculated as the proportion of times a simulated rank for *H. sapiens* was lower than or equal to the associated observed rank over the 200 simulated data sets: *0.01<P≤0.05; **0.005<P≤ 0.01; ***P≤0.005.

| **Species driven extinct** | **Number of remaining species** | ***ED*** | | |  |  |
| --- | --- | --- | --- | --- | --- | --- |
|  |  | **Obs.** | | **Sim.** |  |  |
|  | **mean (SD)** | **Mean**  **(SD)** | | **Mean (SD)**  **[MSD (SSD)]** |  |  |
| **Fritz *et al.* phylogeny** | | | | | | |
| None | 5451 | 2120.07 (19.87) |  | |  |  |
| CR | 5137.95 (4.48) | 1644.48** (16.05) | 2006.65 (17.26)  [**88.07 (10.95)**] | |  |  |
| EN, CR | 4456.16 (5.68) | 254.30*** (6.15) | 1751.01 (15.91)  [**155.10 (13.54)**] | |  |  |
| VU to CR | 3784.84 (4.40) | 80.79*** (4.56) | 1484.64 (16.62)  [**191.07 (15.02)**] | |  |  |
| NT to CR | 3373.46 (3.82) | 80.28*** (3.77) | 1320.25 (17.00)  [**203.88 (15.15)**] | |  |  |
| **Rolland *et al.* phylogeny** | | | | | | |
| None | 5451 | 1332.46 (18.42) | |  | |  |
| CR | 5137.95 (4.48) | 990.55** (14.63) | | 1282.15 (16.04)  [**67.54 (8.46)**] | |  |
| EN, CR | 4456.16 (5.68) | 157.09*** (5.22) | | 1160.97 (14.32)  [**123.67 (10.76)**] | |  |
| VU to CR | 3784.84 (4.40) | 81.08*** (3.57) | | 1023.67 (13.22)  [**154.77 (11.46)**] | |  |
| NT to CR | 3373.46 (3.82) | 81.53*** (3.44) | | 931.42 (13.38)  [**169.10 (10.47)**] | |  |

**Table S1-2.** *Homo sapiens* phylogenetic originality among primates under different scenarios of species extinctions, accounting for missing data. Extinction risk status for species were LC=least concern, NT=near threatened, VU=vulnerable, EN=endangered and CR=critically endangered. Extinction risks for data deficient species were imputed thanks to the missforest algorithm and information on species families, geographical and biological characteristics. Species with missing phylogenetic positions were randomly attached to their family sub-tree or to the sub-tree of the smallest monophyletic group that contains all species from their family. We simulated 500 data sets with phylogenetic positions for all species available in the IUCN Red List and with extinction risk status also for all species. Then for each data set, we considered the *ED* index of phylogenetic originality. Obs. is the observed rank for the originality of *H. sapiens* (where species are ordered from the highest to the lowest originality). Next we drove all CR species to extinction, and performed the same calculations. We repeated this approach when EN and CR species, next VU to CR and then NT to CR species are driven extinct. We compared Obs. with the rank for the originality of *H. sapiens* obtained when permuting (500 times) the IUCN status between all primate species, except *H. sapiens* which conserved the LC status. For each phylogeny, each scenario of species loss, and each originality index, we provide the minimum, median and maximum values of Obs. over the 500 simulated data sets (because the distribution of Obs. values over simulations was sometimes skewed right). The simulated values (Sim.) were bell-shaped; we provide the mean of Sim. and its standard deviation over the 500 simulated data sets. Each data set led to the calculation of one Obs. and 500 Sim. values, the standard deviation of Sim. calculated for each data set is also important as Obs. is compared to the 500 Sim. values per data set. To better describe our results in details, we thus also calculated, for each data set, the standard deviation of Sim. values, which led to 500 standard deviations for which we calculated the mean (MSD) and own standard deviation (SSD). The number of species remaining after each scenario of species loss varied between data sets to account for uncertainties when imputing extinction risk status to data deficient species; this number of species had a bell-shaped distribution, we thus provide its mean and standard deviation over the 500 simulated data sets. In the main text, we chose to present only averaged values over data sets: mean of Obs., mean of Sim. and of its standard deviation (MSD) and mean of the number of species. The P-value was calculated as the proportion of times a simulated rank for *H. sapiens* was lower than or equal to the associated observed rank over the 500 simulated data sets: ^NS^P>0.05; *0.01<P≤0.05; **0.005<P≤ 0.01; ***P≤0.005.

| **Species driven extinct** | **Number of remaining species** | ***ED*** | |  |
| --- | --- | --- | --- | --- |
|  |  | **Obs.** | **Sim.** |  |
|  | **mean (SD)** | **Median**  **[min-max]** | **Mean (SD)**  **[MSD (SSD)]** |  |
| **Pruned Fritz *et al.* phylogeny** | | | | |
| None | 435 | 101 [86,116] |  |  |
| CR | 369.00 (0.66) | 75^NS^ [62,91] | 87.27 (3.85)  [**7.14 (0.96)**] |  |
| EN, CR | 244.00 (0.84) | 6*** [1,11] | 59.39 (2.07)  [**11.58 (0.81)**] |  |
| VU to CR | 156.90 (0.89) | 6*** [4,9] | 39.68 (1.16)  [**11.17 (0.54)**] |  |
| NT to CR | 130.76 (1.12) | 4*** [4,7] | 33.84 (0.95)  [**10.38 (0.44)**] |  |
| **Pruned Rolland *et al.* phylogeny** | | | | |
| None | 435 | 84 [66,103] |  |  |
| CR | 369.00 (0.66) | 62^NS^ [49,79] | 73.93 (4.15)  [**7.75 (0.90)**] |  |
| EN, CR | 244.00 (0.84) | 9*** [4,15] | 52.75 (2.08)  [**10.64 (0.73)**] |  |
| VU to CR | 156.90 (0.89) | 7*** [4,9] | 36.56 (1.13)  [**9.93 (0.50)**] |  |
| NT to CR | 130.76 (1.12) | 3*** [2,5] | 31.64 (0.90)  [**9.28 (0.45)**] |  |
| **Springer *et al.* time tree with autocorrelated rates and hard-bounded constraints** | | | | |
| None | 435 | 46 [33,61] |  |  |
| CR | 369.00 (0.66) | 32* [21,44] | 42.43 (3.44)  [**5.02 (0.63)**] |  |
| EN, CR | 244.00 (0.84) | 5.5*** [1.5,8.5] | 32.81 (1.93)  [**8.08 (0.66)**] |  |
| VU to CR | 156.90 (0.89) | 6* [4,9] | 24.43 (1.17)  [**8.57 (0.53)**] |  |
| NT to CR | 130.76 (1.12) | 4** [2,6] | 21.76 (0.94)  [**8.37 (0.46)**] |  |
| **Springer *et al.* time tree with autocorrelated rates and soft-bounded constraints** | | | | |
| None | 435 | 38 [26,52] |  |  |
| CR | 369.00 (0.66) | 23* [15,37] | 35.53 (3.26)  [**5.26 (0.65)**] |  |
| EN, CR | 244.00 (0.84) | 2*** [1,5] | 27.39 (1.85)  [**7.70 (0.68)**] |  |
| VU to CR | 156.90 (0.89) | 3** [3,6] | 20.70 (1.10)  [**8.02 (0.53)**] |  |
| NT to CR | 130.76 (1.12) | 2*** [2,4] | 18.53 (0.89)  [**7.81 (0.50)**] |  |
| **Springer *et al.* time tree with independent rates and hard-bounded constraints** | | | | |
| None | 435 | 57 [41,74] |  |  |
| CR | 369.00 (0.66) | 40* [30,56] | 53.17 (4.23)  [**5.50 (0.65)**] |  |
| EN, CR | 244.00 (0.84) | 7*** [3,12] | 42.04 (2.70)  [**8.96 (0.73)**] |  |
| VU to CR | 156.90 (0.89) | 8*** [4,11] | 32.45 (1.64)  [**10.10 (0.57)**] |  |
| NT to CR | 130.76 (1.12) | 8** [4,10] | 29.28 (1.37)  [**10.05 (0.52)**] |  |
| **Springer *et al.* time tree with independent rates and soft-bounded constraints** | | | | |
| None | 435 | 56 [40,69] |  |  |
| CR | 369.00 (0.66) | 42^NS^ [27,55] | 51.61 (4.08)  [**5.05 (0.62)**] |  |
| EN, CR | 244.00 (0.84) | 7.5*** [2.5,12.5] | 40.91 (2.56)  [**8.50 (0.69)**] |  |
| VU to CR | 156.90 (0.89) | 7*** [4,11] | 31.57 (1.53)  [**9.68 (0.54)**] |  |
| NT to CR | 130.76 (1.12) | 7** [4,11] | 28.48 (1.26)  [**9.69 (0.49)**] |  |

**Table S1-3.** *Homo sapiens* phylogenetic originality among mammals under different scenarios of species extinctions. **a.** Fritz *et al.* phylogeny; **b.** Rolland *et al.* phylogeny. We considered the *ED* index of phylogenetic originality. LC=least concern; NT=near threatened; VU=vulnerable; EN=endangered; CR=critically endangered. Species for which limited biological information was available (data deficient species, DD) were treated by two extreme scenarios: as LC, as CR. *N* is the number of species. Obs. is the observed rank for the originality of *H. sapiens* (where species are ordered from the highest to the lowest originality); Sim. are the simulated ranks obtained for *H. sapiens* after permuting the IUCN status of non-human mammals (500 times); SD is the standard deviation of the simulated ranks. The P-value was calculated as the proportion of times a simulated rank for *H. sapiens* was lower than or equal to the observed rank: **0.005<P≤ 0.01; ***P≤0.005.

**a**

| **Species driven extinct** | ***N*** | ***ED*** | |  |
| --- | --- | --- | --- | --- |
|  |  | **Obs.** | **Mean Sim. (SD)** |  |
| None | 4854 | 1989 |  |  |
| **Treating DD species as LC** | | | | |
| CR | 4704 | 1593*** | 1927.70 (55.28) |  |
| EN, CR | 4313 | 86*** | 1776.17 (106.02) |  |
| VU, EN, CR | 3857 | 85*** | 1583.44 (146.81) |  |
| NT to CR | 3534 | 75*** | 1444.92 (161.01) |  |
| **Treating DD species as CR** | | | | |
| CR | 4118 | 1444* | 1693.11 (132.99) |  |
| EN, CR | 3727 | 85*** | 1526.22 (156.38) |  |
| VU, EN, CR | 3271 | 80*** | 1340.39 (167.08) |  |
| NT to CR | 2948 | 82*** | 1193.99 (167.81) |  |

**b**

| **Species driven extinct** | ***N*** | ***ED*** | | | |  |  |
| --- | --- | --- | --- | --- | --- | --- | --- |
|  |  | **Obs.** | **Mean Sim. (SD)** | | |  |  |
| None | 4854 | 2954 | |  |  | |  |
| **Treating DD species as LC** | | | | | | | |
| CR | 4704 | 926*** | 1203.04 (38.47) | | |  |  |
| EN, CR | 4313 | 84*** | 1139.08 (82.47) | | |  |  |
| VU, EN, CR | 3857 | 77*** | 1047.05 (118.09) | | |  |  |
| NT to CR | 3534 | 76*** | 980.33 (135.15) | | |  |  |
| **Treating DD species as CR** | | | | | | | |
| CR | 4118 | 911*** | 1808.72 (42.53) | | |  |  |
| EN, CR | 3727 | 96*** | 1066.81 (97.11) | | |  |  |
| VU, EN, CR | 3271 | 84*** | 968.59 (119.40) | | |  |  |
| NT to CR | 2948 | 83*** | 877.51 (138.70) | | |  |  |

**Table S1-4.** *Homo sapiens* phylogenetic originality among primates under different scenarios of species extinctions using **a**. Pruned Fritz *et al.* phylogeny; **b.** Pruned Rolland *et al.* phylogeny; **c.** Springer *et al.* time tree with autocorrelated rates and hard-bounded constraints; **d.** Springer *et al.* primate time tree with autocorrelated rates and soft-bounded constraints; **e.** Springer *et al.* primate time tree with independent rates and hard-bounded constraints; **f.** Springer *et al.* primate time tree with independent rates and soft-bounded constraints.We considered the *ED* index of phylogenetic originality. LC=least concern; NT=near threatened; VU=vulnerable; EN=endangered; CR=critically endangered. Species for which limited biological information was available (data deficient species, DD) were treated by two extreme scenarios: as LC, as CR. N is the number of species. Obs. is the observed rank for the originality of *H. sapiens* (where species are ordered from the highest to the lowest originality); Sim. are the simulated ranks obtained for *H. sapiens* after permuting the IUCN status of non-human primates (1000 times). The P-value was calculated as the proportion of times a simulated rank for *H. sapiens* was lower than or equal to the observed rank: NSP>0.05; *0.01<P≤ 0.05; **0.005<P≤0.01; ***P≤0.005.

**a**

| **Species driven extinct** | ***N*** | ***ED*** | | | |  |  |
| --- | --- | --- | --- | --- | --- | --- | --- |
|  |  | **Obs.** | **Mean Sim. (SD)** | | |  |  |
| None | 331 | 88 | |  |  | |  |
| **Treating DD species as LC** | | | | | | | |
| CR | 291 | 72^NS^ | 77.50 (3.92) | | |  |  |
| EN, CR | 208 | 3*** | 55.57 (8.05) | | |  |  |
| VU, EN, CR | 142 | 6*** | 38.84 (7.89) | | |  |  |
| NT, VU, EN, CR | 120 | 3*** | 32.79 (7.65) | | |  |  |
| **Treating DD species as CR** | | | | | | | |
| CR | 281 | 69^NS^ | 74.63 (4.68) | | |  |  |
| EN, CR | 198 | 4*** | 53.12 (7.80) | | |  |  |
| VU, EN, CR | 132 | 6*** | 35.59 (7.81) | | |  |  |
| NT, VU, EN, CR | 110 | 5*** | 30.47 (7.49) | | |  |  |

**b**

| **Species driven extinct** | ***N*** | ***ED*** | | | |  |  |
| --- | --- | --- | --- | --- | --- | --- | --- |
|  |  | **Obs.** | **Mean Sim. (SD)** | | |  |  |
| None | 331 | 136 | |  |  | |  |
| **Treating DD species as LC** | | | | | | | |
| CR | 291 | 55^NS^ | 60.63 (4.88) | | |  |  |
| EN, CR | 208 | 4*** | 46.86 (7.84) | | |  |  |
| VU, EN, CR | 142 | 6*** | 34.46 (7.53) | | |  |  |
| NT, VU, EN, CR | 120 | 3*** | 29.69 (7.37) | | |  |  |
| **Treating DD species as CR** | | | | | | | |
| CR | 281 | 52^NS^ | 59.17 (5.71) | | |  |  |
| EN, CR | 198 | 5*** | 45.43 (7.74) | | |  |  |
| VU, EN, CR | 132 | 6*** | 32.22 (7.11) | | |  |  |
| NT, VU, EN, CR | 110 | 3*** | 27.85 (6.79) | | |  |  |

**c**

| **Species driven extinct** | ***N*** | ***ED*** | | | |  |  |
| --- | --- | --- | --- | --- | --- | --- | --- |
|  |  | **Obs.** | **Mean Sim. (SD)** | | |  |  |
| None | 340 | 40 | |  |  | |  |
| **Treating DD species as LC** | | | | | | | |
| CR | 294 | 25* | 30.62 (3.24) | | |  |  |
| EN, CR | 194 | 4.5*** | 26.19 (6.12) | | |  |  |
| VU, EN, CR | 126 | 5*** | 21.30 (7.10) | | |  |  |
| NT, VU, EN, CR | 105 | 5* | 19.57 (7.21) | | |  |  |
| **Treating DD species as CR** | | | | | | | |
| CR | 284 | 24* | 30.42 (3.79) | | |  |  |
| EN, CR | 184 | 7.5** | 25.78 (6.32) | | |  |  |
| VU, EN, CR | 116 | 6* | 20.20 (7.21) | | |  |  |
| NT, VU, EN, CR | 95 | 4*** | 18.63 (6.96) | | |  |  |

**d**

| **Species driven extinct** | ***N*** | ***ED*** | | | |  |
| --- | --- | --- | --- | --- | --- | --- |
|  |  | **Obs.** | **Mean Sim. (SD)** | | |  |
| None | 340 | 26 | |  |  | |
| **Treating DD species as LC** | | | | | |  |
| CR | 294 | 21^NS^ | 25.67 (3.04) | | |  |
| EN, CR | 194 | 3*** | 22.24 (5.96) | | |  |
| VU, EN, CR | 126 | 4** | 18.37 (6.73) | | |  |
| NT, VU, EN, CR | 105 | 4* | 16.95 (6.80) | | |  |
| **Treating DD species as CR** | | | | | |  |
| CR | 284 | 20^NS^ | 25.53 (3.51) | | |  |
| EN, CR | 184 | 5** | 21.85 (6.15) | | |  |
| VU, EN, CR | 116 | 4* | 17.72 (6.65) | | |  |
| NT, VU, EN, CR | 95 | 3** | 16.10 (6.74) | | |  |

**e**

| **Species driven extinct** | ***N*** | ***ED*** | | | |  |
| --- | --- | --- | --- | --- | --- | --- |
|  |  | **Obs.** | **Mean Sim. (SD)** | | |  |
| None | 340 | 102 | |  |  | |
| **Treating DD species as LC** | | | | | |  |
| CR | 294 | 30^NS^ | 35.59 (3.70) | | |  |
| EN, CR | 194 | 4.5*** | 33.07 (7.25) | | |  |
| VU, EN, CR | 126 | 6*** | 27.82 (8.62) | | |  |
| NT, VU, EN, CR | 105 | 6*** | 25.90 (8.94) | | |  |
| **Treating DD species as CR** | | | | | |  |
| CR | 284 | 27* | 35.55 (4.35) | | |  |
| EN, CR | 184 | 9.5** | 32.53 (7.56) | | |  |
| VU, EN, CR | 116 | 7*** | 26.94 (8.72) | | |  |
| NT, VU, EN, CR | 95 | 7** | 24.37 (8.66) | | |  |

**f**

| **Species driven extinct** | ***N*** | ***ED*** | | | |  |
| --- | --- | --- | --- | --- | --- | --- |
|  |  | **Obs.** | **Mean Sim. (SD)** | | |  |
| None | 340 | 122 | |  |  | |
| **Treating DD species as LC** | | | | | |  |
| CR | 294 | 28* | 34.03 (3.40) | | |  |
| EN, CR | 194 | 6.5*** | 31.88 (6.81) | | |  |
| VU, EN, CR | 126 | 8*** | 27.04 (8.28) | | |  |
| NT, VU, EN, CR | 105 | 6*** | 25.19 (8.54) | | |  |
| **Treating DD species as CR** | | | | | |  |
| CR | 284 | 25* | 34.04 (4.07) | | |  |
| EN, CR | 184 | 9.5*** | 31.44 (7.06) | | |  |
| VU, EN, CR | 116 | 7*** | 26.09 (8.32) | | |  |
| NT, VU, EN, CR | 95 | 7*** | 23.72 (8.23) | | |  |

**Table S1-5.** *Homo sapiens* phylogenetic originality among mammals under different scenarios of species extinctions. **a.** Fritz *et al.* phylogeny; **b.** Rolland *et al.* phylogeny. The legend is similar as that of Table S1-3. Compared to Table S1-3, we used here phylogenetically controlled permutations.

**a**

| **Species driven extinct** | ***N*** | ***ED*** | | | |
| --- | --- | --- | --- | --- | --- |
|  |  | **Obs.** | **Mean Sim. (SD)** | | |
| None | 4854 | 1989 | |  |  |
| **Treating DD species as LC** | | | | | |
| CR | 4704 | 1593*** | 1926.66 (54.35) | | |
| EN, CR | 4313 | 86*** | 1766.19 (105.81) | | |
| VU, EN, CR | 3857 | 85*** | 1573.02 (133.46) | | |
| NT to CR | 3534 | 75*** | 1442.82 (134.33) | | |
| **Treating DD species as CR** | | | | | |
| CR | 4118 | 1444* | 1696.49 (120.19) | | |
| EN, CR | 3727 | 85*** | 1527.56 (139.83) | | |
| VU, EN, CR | 3271 | 80*** | 1331.03 (149.83) | | |
| NT to CR | 2948 | 82*** | 1200.47 (140.89) | | |

**b**

| **Species driven extinct** | ***N*** | ***ED*** | | | |
| --- | --- | --- | --- | --- | --- |
|  |  | **Obs.** | **Mean Sim. (SD)** | | |
| None | 4854 | 2954 | |  |  |
| **Treating DD species as LC** | | | | | |
| CR | 4704 | 926*** | 1203.02 (35.17) | | |
| EN, CR | 4313 | 84*** | 1136.07 (80.61) | | |
| VU, EN, CR | 3857 | 77*** | 1035.21 (124.97) | | |
| NT to CR | 3534 | 76*** | 965.11 (133.45) | | |
| **Treating DD species as CR** | | | | | |
| CR | 4118 | 911^NS^ | 1093.11 (99.86) | | |
| EN, CR | 3727 | 96*** | 1011.56 (130.33) | | |
| VU, EN, CR | 3271 | 84*** | 898.33 (156.62) | | |
| NT to CR | 2948 | 83*** | 820.23 (157.62) | | |

**Table S1-6.** *Homo sapiens* phylogenetic originality among primates under different scenarios of species extinctions using **a**. Pruned Fritz *et al.* phylogeny; **b.** Pruned Rolland *et al.* phylogeny; **c.** Springer *et al.* time tree with autocorrelated rates and hard-bounded constraints; **d.** Springer *et al.* primate time tree with autocorrelated rates and soft-bounded constraints; **e.** Springer *et al.* primate time tree with independent rates and hard-bounded constraints; **f.** Springer *et al.* primate time tree with independent rates and soft-bounded constraints. The legend is similar as that of Table S1-4. Compared to Table S1-4, we used here phylogenetically controlled permutations.

**a**

| **Species driven extinct** | ***N*** | ***ED*** | | | |  |  |
| --- | --- | --- | --- | --- | --- | --- | --- |
|  |  | **Obs.** | **Mean Sim. (SD)** | | |  |  |
| None | 331 | 88 | |  |  | |  |
| **Treating DD species as LC** | | | | | | | |
| CR | 291 | 72^NS^ | 76.35 (4.74) | | |  |  |
| EN, CR | 208 | 3*** | 54.08 (7.06) | | |  |  |
| VU, EN, CR | 142 | 6*** | 34.52 (6.46) | | |  |  |
| NT, VU, EN, CR | 120 | 3*** | 29.16 (6.68) | | |  |  |
| **Treating DD species as CR** | | | | | | | |
| CR | 281 | 69^NS^ | 73.82 (5.25) | | |  |  |
| EN, CR | 198 | 4*** | 51.10 (6.97) | | |  |  |
| VU, EN, CR | 132 | 6*** | 31.56 (6.19) | | |  |  |
| NT, VU, EN, CR | 110 | 5*** | 26.09 (6.51) | | |  |  |

**b**

| **Species driven extinct** | ***N*** | ***ED*** | | | |  |  |
| --- | --- | --- | --- | --- | --- | --- | --- |
|  |  | **Obs.** | **Mean Sim. (SD)** | | |  |  |
| None | 331 | 136 | |  |  | |  |
| **Treating DD species as LC** | | | | | | | |
| CR | 291 | 55^NS^ | 62.54 (5.22) | | |  |  |
| EN, CR | 208 | 4*** | 46.56 (6.92) | | |  |  |
| VU, EN, CR | 142 | 6*** | 30.61 (5.94) | | |  |  |
| NT, VU, EN, CR | 120 | 3*** | 26.49 (5.98) | | |  |  |
| **Treating DD species as CR** | | | | | | | |
| CR | 281 | 52^NS^ | 60.80 (5.70) | | |  |  |
| EN, CR | 198 | 5*** | 44.42 (6.84) | | |  |  |
| VU, EN, CR | 132 | 6*** | 28.41 (5.63) | | |  |  |
| NT, VU, EN, CR | 110 | 3*** | 24.16 (5.54) | | |  |  |

**c**

| **Species driven extinct** | ***N*** | ***ED*** | | | |  |  |
| --- | --- | --- | --- | --- | --- | --- | --- |
|  |  | **Obs.** | **Mean Sim. (SD)** | | |  |  |
| None | 340 | 40 | |  |  | |  |
| **Treating DD species as LC** | | | | | | | |
| CR | 294 | 25* | 30.40 (3.26) | | |  |  |
| EN, CR | 194 | 4.5*** | 27.57 (6.75) | | |  |  |
| VU, EN, CR | 126 | 5** | 24.00 (7.94) | | |  |  |
| NT, VU, EN, CR | 105 | 5** | 21.18 (7.39) | | |  |  |
| **Treating DD species as CR** | | | | | | | |
| CR | 284 | 24^NS^ | 29.21 (3.61) | | |  |  |
| EN, CR | 184 | 7.5* | 26.44 (6.73) | | |  |  |
| VU, EN, CR | 116 | 6* | 22.30 (7.47) | | |  |  |
| NT, VU, EN, CR | 95 | 4*** | 19.29 (6.96) | | |  |  |

**d**

| **Species driven extinct** | ***N*** | ***ED*** | | | |  |  |
| --- | --- | --- | --- | --- | --- | --- | --- |
|  |  | **Obs.** | **Mean Sim. (SD)** | | |  |  |
| None | 340 | 56 | |  |  | |  |
| **Treating DD species as LC** | | | | | | | |
| CR | 294 | 21* | 26.26 (2.78) | | |  |  |
| EN, CR | 194 | 3*** | 23.37 (6.21) | | |  |  |
| VU, EN, CR | 126 | 4*** | 20.94 (6.95) | | |  |  |
| NT, VU, EN, CR | 105 | 4*** | 18.67 (7.05) | | |  |  |
| **Treating DD species as CR** | | | | | | | |
| CR | 284 | 20^NS^ | 25.16 (2.97) | | |  |  |
| EN, CR | 184 | 5*** | 22.20 (6.03) | | |  |  |
| VU, EN, CR | 116 | 4** | 19.64 (6.98) | | |  |  |
| NT, VU, EN, CR | 95 | 3** | 17.19 (6.92) | | |  |  |

**e**

| **Species driven extinct** | ***N*** | ***ED*** | | | |  |  |
| --- | --- | --- | --- | --- | --- | --- | --- |
|  |  | **Obs.** | **Mean Sim. (SD)** | | |  |  |
| None | 340 | 102 | |  |  | |  |
| **Treating DD species as LC** | | | | | | | |
| CR | 294 | 30^NS^ | 35.45 (3.77) | | |  |  |
| EN, CR | 194 | 4.5*** | 37.01 (8.29) | | |  |  |
| VU, EN, CR | 126 | 6*** | 32.51 (8.10) | | |  |  |
| NT, VU, EN, CR | 105 | 6*** | 28.54 (7.81) | | |  |  |
| **Treating DD species as CR** | | | | | | | |
| CR | 284 | 27* | 35.16 (4.17) | | |  |  |
| EN, CR | 184 | 9.5** | 36.41 (8.74) | | |  |  |
| VU, EN, CR | 116 | 7*** | 30.74 (7.96) | | |  |  |
| NT, VU, EN, CR | 95 | 7*** | 26.25 (7.55) | | |  |  |

**f**

| **Species driven extinct** | ***N*** | ***ED*** | |  |
| --- | --- | --- | --- | --- |
|  |  | **Obs.** | **Mean Sim. (SD)** |  |
| None | 340 | 122 |  |  |
| **Treating DD species as LC** | | | | |
| CR | 294 | 28* | 34.10 (3.30) |  |
| EN, CR | 194 | 6.5*** | 36.37 (5.56) |  |
| VU, EN, CR | 126 | 8*** | 31.15 (7.80) |  |
| NT, VU, EN, CR | 105 | 6*** | 27.16 (7.38) |  |
| **Treating DD species as CR** | | | | |
| CR | 284 | 25* | 33.64 (3.74) |  |
| EN, CR | 184 | 9.5*** | 35.26 (7.90) |  |
| VU, EN, CR | 116 | 7*** | 29.03 (7.62) |  |
| NT, VU, EN, CR | 95 | 7** | 24.90 (7.11) |  |

Table S1-7. Ordinal phylogenetic model in a Bayesian MCMC framework testing the potential effects of 16 specified explanatory variables on species extinction risks with the following phylogenies: mamF= Fritz et al. phylogeny for mammals; F= pruned Fritz et al. phylogeny; R= pruned Rolland et al. phylogeny; AS= Springer et al. primate phylogeny with autocorrelated rates and soft-bounded constraints; IH= Springer et al. primate phylogeny with independent rates and hard-bounded constraints; IS= Springer et al. primate phylogeny with independent rates and soft-bounded constraints.

| *Phylogeny* | *Variable^a^* | *Posterior mean* | CI | pMCMC |
| --- | --- | --- | --- | --- |
| mamF | Distance to *H. sapiens^b^* | -0.221 | [-0.971; 0.299] | 0.560 |
|  | Originality | -0.034 | [-0.261; 0.143] | 0.752 |
|  | Nb of known threats*^c^* | 1.116 | [0.908; 1.340] | <<0.001 |
|  | Body mass | 0.946 | [0.624; 1.326] | <<0.001 |
|  | Geographic range size | -1.310 | [-1.551; -1.019] | <<0.001 |
|  | Diet breadth | 0.004 | [-0.183; 0.172] | 0.984 |
|  | Habitat breadth | 0.091 | [-0.059; 0.263] | 0.296 |
|  | Litter size | -0.099 | [-0.349; 0.108] | 0.464 |
|  | Trophic level | 0.323 | [0.088; 0.552] | 0.016 |
|  | Med. latitude (deg. 1)*^d^* | 0.108 | [-0.133; 0.318] | 0.344 |
|  | Med. latitude (deg. 2)*^d^* | 0.088 | [-0.140; 0.309] | 0.440 |
|  | Med. longitude (deg. 1)*^d^* | 0.254 | [0.038; 0.473] | 0.040 |
|  | Med. longitude (deg. 2)*^d^* | -0.087 | [-0.326; 0.107] | 0.424 |
|  | Human pop. density*^e^* | 0.098 | [-0.020; 0.214] | 0.160 |
|  | Human pop. change*^f^* | 0.074 | [-0.109; 0.250] | 0.320 |
|  | Precipitation | 0.037 | [-0.135; 0.270] | 0.752 |
| F | Distance to *H. sapiens^b^* | 0.084 | [-3.540; 3.895] | 0.962 |
|  | Originality | 0.311 | [-2.232; 2.681] | 0.782 |
|  | Nb of known threats*^c^* | 3.941 | [1.290; 6.610] | <<0.001 |
|  | Body mass | 1.707 | [-1.289; 4.722] | 0.255 |
|  | Geographic range size | -4.152 | [-6.712; -1.830] | <<0.001 |
|  | Diet breadth | -1.059 | [-3.057; 0.732] | 0.224 |
|  | Habitat breadth | 1.013 | [-0.621; 2.892] | 0.212 |
|  | Litter size | 0.060 | [-2.390; 2.539] | 0.935 |
|  | Trophic level | -0.783 | [-2.948; 1.049] | 0.433 |
|  | Med. latitude (deg. 1)*^d^* | 0.066 | [-2.225; 2.264] | 0.960 |
|  | Med. latitude (deg. 2)*^d^* | -0.512 | [-2.432; 1.221] | 0.545 |
|  | Med. longitude (deg. 1)*^d^* | -0.028 | [-3.153; 3.081] | 0.980 |
|  | Med. longitude (deg. 2)*^d^* | -0.626 | [-3.071; 1.585] | 0.586 |
|  | Human pop. density*^e^* | 1.259 | [-0.273; 2.937] | 0.088 |
|  | Human pop. change*^f^* | -0.415 | [-2.400; 1.550] | 0.670 |
|  | Precipitation | -0.894 | [-2.609; 0.659] | 0.270 |
| R | Distance to *H. sapiens^b^* | -0.088 | [-3.581; 3.618] | 0.960 |
|  | Originality | 1.629 | [-0.542; 4.198] | 0.140 |
|  | Nb of known threats*^c^* | 2.939 | [0.818; 5.555] | <<0.001 |
|  | Body mass | 1.807 | [-1.158; 4.696] | 0.208 |
|  | Geographic range size | -3.979 | [-6.601; -1.691] | <<0.001 |
|  | Diet breadth | -1.356 | [-3.360; 0.324] | 0.105 |
|  | Habitat breadth | 1.064 | [-0.574; 2.819] | 0.165 |
|  | Litter size | 0.626 | [-1.834; 2.795] | 0.526 |
|  | Trophic level | -0.612 | [-2.578; 1.186] | 0.505 |
|  | Med. latitude (deg. 1)*^d^* | 0.067 | [-1.992; 2.315] | 0.950 |
|  | Med. latitude (deg. 2)*^d^* | -0.382 | [-2.146; 1.316] | 0.604 |
|  | Med. longitude (deg. 1)*^d^* | -0.191 | [-3.292; 2.716] | 0.897 |
|  | Med. longitude (deg. 2)*^d^* | -0.382 | [-2.635; 1.793] | 0.749 |
|  | Human pop. density*^e^* | 1.202 | [-0.142; 2.996] | 0.078 |
|  | Human pop. change*^f^* | -0.435 | [-2.346; 1.418] | 0.606 |
|  | Precipitation | -1.077 | [-2.840; 0.490] | 0.175 |
| AS | Distance to *H. sapiens^b^* | -0.052 | [-3.360; 3.538] | 0.956 |
|  | Originality | 1.356 | [-0.442; 3.836] | 0.132 |
|  | Nb of known threats*^c^* | 2.711 | [0.417; 5.567] | 0.002 |
|  | Body mass | 1.507 | [-1.160; 4.303] | 0.227 |
|  | Geographic range size | -3.77 | [-6.347; -1.487] | <0.001 |
|  | Diet breadth | -0.562 | [-2.510; 0.924] | 0.476 |
|  | Habitat breadth | 0.728 | [-0.782; 2.410] | 0.288 |
|  | Litter size | -0.129 | [-2.491; 1.980] | 0.972 |
|  | Trophic level | -0.409 | [-2.420; 1.375] | 0.607 |
|  | Med. latitude (deg. 1)*^d^* | 0.585 | [-1.374; 2.818] | 0.544 |
|  | Med. latitude (deg. 2)*^d^* | -0.688 | [-2.527; 0.795] | 0.328 |
|  | Med. longitude (deg. 1)*^d^* | -0.159 | [-3.131; 2.594] | 0.952 |
|  | Med. longitude (deg. 2)*^d^* | -0.732 | [-3.078; 1.269] | 0.494 |
|  | Human pop. density*^e^* | 1.221 | [-0.245; 3.234] | 0.112 |
|  | Human pop. change*^f^* | -0.294 | [-2.091; 1.319] | 0.726 |
|  | Precipitation | -0.485 | [-2.066; 0.859] | 0.530 |
| IH | Distance to *H. sapiens^b^* | -0.077 | [-3.055; 3.327] | 0.935 |
|  | Originality | 0.969 | [-0.688; 3.241] | 0.168 |
|  | Nb of known threats*^c^* | 2.088 | [0.243; 5.259] | 0.001 |
|  | Body mass | 1.414 | [-0.955; 3.889] | 0.157 |
|  | Geographic range size | -3.068 | [-5.576; -1.239] | <<0.001 |
|  | Diet breadth | -0.392 | [-2.085; 0.797] | 0.543 |
|  | Habitat breadth | 0.498 | [-0.634; 1.982] | 0.310 |
|  | Litter size | -0.056 | [-2.387; 1.500] | 0.823 |
|  | Trophic level | -2.445 | [-1.748; 1.346] | 0.634 |
|  | Med. latitude (deg. 1)*^d^* | 0.443 | [-1.057; 2.484] | 0.591 |
|  | Med. latitude (deg. 2)*^d^* | -0.581 | [-2.200; 0.532] | 0.289 |
|  | Med. longitude (deg. 1)*^d^* | -0.062 | [-2.871; 2.170] | 0.950 |
|  | Med. longitude (deg. 2)*^d^* | -0.495 | [-2.648; 1.079] | 0.595 |
|  | Human pop. density*^e^* | 0.805 | [-0.402; 2.679] | 0.229 |
|  | Human pop. change*^f^* | -0.186 | [-1.846; 1.150] | 0.802 |
|  | Precipitation | -0.245 | [-1.690; 0.790] | 0.750 |
| IS | Distance to *H. sapiens^b^* | -0.085 | [-2.954; 3.046] | 0.928 |
|  | Originality | 0.963 | [-0.572; 2.836] | 0.114 |
|  | Nb of known threats*^c^* | 1.764 | [0.218; 4.715] | 0.001 |
|  | Body mass | 1.449 | [-0.555; 3.690] | 0.109 |
|  | Geographic range size | -2.875 | [-5.152; -1.116] | <<0.001 |
|  | Diet breadth | -0.342 | [-1.752; 0.740] | 0.541 |
|  | Habitat breadth | 0.444 | [-0.571; 1.754] | 0.307 |
|  | Litter size | 0.046 | [-2.057; 1.438] | 0.714 |
|  | Trophic level | -0.258 | [-1.796; 1.048] | 0.575 |
|  | Med. latitude (deg. 1)*^d^* | 0.401 | [-0.979; 2.279] | 0.577 |
|  | Med. latitude (deg. 2)*^d^* | -0.540 | [-1.952; 0.484] | 0.262 |
|  | Med. longitude (deg. 1)*^d^* | -0.019 | [-2.391; 2.203] | 0.898 |
|  | Med. longitude (deg. 2)*^d^* | -0.414 | [-2.283; 1.046] | 0.624 |
|  | Human pop. density*^e^* | 0.654 | [-0.371; 2.399] | 0.252 |
|  | Human pop. change*^f^* | -0.139 | [-1.502; 0.988] | 0.856 |
|  | Precipitation | -0.190 | [-1.363; 0.784] | 0.803 |

*^a^ See the Methods section and the database PanTHERIA, Jones et al. 2009, for details on each variable (all variables were scaled to a mean of 0 and a variance of 1); ^b^ in Million years of evolution; ^c^ number of known threat types affecting a species; ^d^ Median (Med.) latitudinal extent of each species range and median longitudinal extent of each species range, each expressed as orthogonal polynomials of degree (deg.) 1 and 2; ^e^ human population density; ^f^ mean rate of increase in human population density.*

Table S1-8. Ordinal phylogenetic model in a Bayesian MCMC framework testing the potential effects of 16 specified explanatory variables on species extinction risks with the following phylogenies: mamF= Fritz et al. phylogeny for mammals; F= pruned Fritz et al. phylogeny; R= pruned Rolland et al. phylogeny; AS= Springer et al. primate phylogeny with autocorrelated rates and soft-bounded constraints; IH= Springer et al. primate phylogeny with independent rates and hard-bounded constraints; IS= Springer et al. primate phylogeny with independent rates and soft-bounded constraints. Compared to Table S1-8, here we removed threatened species not listed under criterion A of the IUCN Red List.

| *Phylogeny* | *Variable^a^* | *Posterior mean* | CI | pMCMC |
| --- | --- | --- | --- | --- |
| mamF | Distance to *H. sapiens^b^* | -0.206 | [-0.991; 0.519] | 0.600 |
|  | Originality | -0.058 | [-0.337; 0.157] | 0.584 |
|  | Nb of known threats*^c^* | 1.068 | [0.849; 1.312] | <<0.001 |
|  | Body mass | 1.274 | [0.900; 1.669] | <<0.001 |
|  | Geographic range size | -1.258 | [-1.616; -0.999] | <<0.001 |
|  | Diet breadth | 0.059 | [-0.124; 0.266] | 0.552 |
|  | Habitat breadth | 0.198 | [0.004; 0.417] | 0.056 |
|  | Litter size | -0.189 | [-0.525; 0.094] | 0.208 |
|  | Trophic level | 0.311 | [-0.003; 0.575] | 0.048 |
|  | Med. latitude (deg. 1)*^d^* | 0.192 | [-0.057; 0.451] | 0.112 |
|  | Med. latitude (deg. 2)*^d^* | 0.116 | [-0.113; 0.342] | 0.360 |
|  | Med. longitude (deg. 1)*^d^* | 0.196 | [-0.104; 0.519] | 0.168 |
|  | Med. longitude (deg. 2)*^d^* | -0.175 | [-0.403; 0.092] | 0.168 |
|  | Human pop. density*^e^* | -0.009 | [-0.185; 0.186] | 0.864 |
|  | Human pop. change*^f^* | -0.117 | [-0.372; 0.122] | 0.320 |
|  | Precipitation | 0.217 | [-0.006; 0.423] | 0.056 |
| F | Distance to *H. sapiens^b^* | 0.093 | [-3.731; 3.722] | 0.960 |
|  | Originality | 1.128 | [-1.321; 3.784] | 0.366 |
|  | Nb of known threats*^c^* | 4.375 | [1.933; 7.053] | <<0.001 |
|  | Body mass | 2.168 | [-0.873; 5.219] | 0.156 |
|  | Geographic range size | -3.782 | [-6.284; -1.370] | 0.001 |
|  | Diet breadth | -0.106 | [-2.126; 1.899] | 0.918 |
|  | Habitat breadth | 0.564 | [-1.277; 2.429] | 0.538 |
|  | Litter size | -0.062 | [-2.729; 2.513] | 0.971 |
|  | Trophic level | 0.666 | [-2.739; 1.402] | 0.519 |
|  | Med. latitude (deg. 1)*^d^* | -0.250 | [-2.828; 2.607] | 0.850 |
|  | Med. latitude (deg. 2)*^d^* | -1.028 | [-3.432; 1.364] | 0.392 |
|  | Med. longitude (deg. 1)*^d^* | -0.219 | [-3.375; 3.024] | 0.900 |
|  | Med. longitude (deg. 2)*^d^* | -0.971 | [-3.456; 1.478] | 0.428 |
|  | Human pop. density*^e^* | 2.644 | [0.598; 4.844] | 0.004 |
|  | Human pop. change*^f^* | 0.377 | [-1.846; 2.422] | 0.719 |
|  | Precipitation | 0.034 | [-1.831; 1.767] | 0.940 |
| R | Distance to *H. sapiens^b^* | 0.024 | [-3.545; 3.674] | 0.975 |
|  | Originality | 2.809 | [0.383; 5.190] | 0.017 |
|  | Nb of known threats*^c^* | 4.165 | [1.844; 6.621] | <<0.001 |
|  | Body mass | 2.169 | [-0.597; 5.155] | 0.135 |
|  | Geographic range size | -3.594 | [-5.999; -1.349] | <0.001 |
|  | Diet breadth | -0.365 | [-2.211; 1.379] | 0.680 |
|  | Habitat breadth | 0.558 | [-1.255; 2.278] | 0.522 |
|  | Litter size | 0.450 | [-1.871; 2.789] | 0.671 |
|  | Trophic level | -0.561 | [-2.512; 1.125] | 0.542 |
|  | Med. latitude (deg. 1)*^d^* | 0.023 | [-2.430; 2.555] | 0.987 |
|  | Med. latitude (deg. 2)*^d^* | -1.135 | [-3.320; 1.070] | 0.287 |
|  | Med. longitude (deg. 1)*^d^* | -0.273 | [-3.487; 2.733] | 0.860 |
|  | Med. longitude (deg. 2)*^d^* | -0.906 | [-3.211; 1.394] | 0.420 |
|  | Human pop. density*^e^* | 2.590 | [0.557; 4.601] | 0.003 |
|  | Human pop. change*^f^* | 0.180 | [-1.716; 2.054] | 0.855 |
|  | Precipitation | 0.245 | [-1.353; 1.870] | 0.733 |
| AS | Distance to *H. sapiens^b^* | 0.098 | [-3.490; 3.713] | 0.952 |
|  | Originality | 1.480 | [-0.803; 3.913] | 0.189 |
|  | Nb of known threats*^c^* | 4.014 | [1.353; 6.801] | <<0.001 |
|  | Body mass | 1.714 | [-1.174; 4.809] | 0.230 |
|  | Geographic range size | -3.798 | [-6.319; -1.462] | <0.001 |
|  | Diet breadth | -0.031 | [-1.943; 1.838] | 0.983 |
|  | Habitat breadth | 0.315 | [-1.636; 2.148] | 0.710 |
|  | Litter size | -0.097 | [-2.751; 2.519] | 0.979 |
|  | Trophic level | -0.194 | [-2.219; 1.916] | 0.855 |
|  | Med. latitude (deg. 1)*^d^* | 0.203 | [-2.420; 2.951] | 0.889 |
|  | Med. latitude (deg. 2)*^d^* | -0.722 | [-3.070; 1.516] | 0.512 |
|  | Med. longitude (deg. 1)*^d^* | -0.297 | [-3.465; 2.896] | 0.857 |
|  | Med. longitude (deg. 2)*^d^* | -1.051 | [-3.616; 1.224] | 0.374 |
|  | Human pop. density*^e^* | 1.907 | [0.134; 3.932] | 0.025 |
|  | Human pop. change*^f^* | -0.002 | [-1.898; 1.912] | 0.988 |
|  | Precipitation | 0.230 | [-1.515; 1.962] | 0.739 |
| IH | Distance to *H. sapiens^b^* | 0.101 | [-3.611; 3.601] | 0.947 |
|  | Originality | 1.327 | [-0.938; 3.873] | 0.224 |
|  | Nb of known threats*^c^* | 3.780 | [1.146; 6.725] | <0.001 |
|  | Body mass | 1.670 | [-1.160; 4.515] | 0.229 |
|  | Geographic range size | -3.427 | [-5.728; -1.138] | <0.001 |
|  | Diet breadth | 0.004 | [-1.828; 1.729] | 0.970 |
|  | Habitat breadth | 0.220 | [-1.455; 2.160] | 0.771 |
|  | Litter size | -0.117 | [-2.704; 2.325] | 0.998 |
|  | Trophic level | -0.027 | [-1.935; 1.883] | 0.965 |
|  | Med. latitude (deg. 1)*^d^* | 0.288 | [-2.186; 2.925] | 0.817 |
|  | Med. latitude (deg. 2)*^d^* | -0.707 | [-2.892; 1.438] | 0.483 |
|  | Med. longitude (deg. 1)*^d^* | -0.315 | [-3.438; 2.468] | 0.843 |
|  | Med. longitude (deg. 2)*^d^* | -0.927 | [-3.315; 1.201] | 0.397 |
|  | Human pop. density*^e^* | 1.622 | [-0.201; 3.578] | 0.037 |
|  | Human pop. change*^f^* | -0.005 | [-1.805; 1.649] | 0.997 |
|  | Precipitation | 0.298 | [-1.443; 1.890] | 0.668 |
| IS | Distance to *H. sapiens^b^* | 0.086 | [-3.507; 3.787] | 0.967 |
|  | Originality | 1.344 | [-0.881; 3.970] | 0.216 |
|  | Nb of known threats*^c^* | 3.721 | [1.013; 6.540] | <<0.001 |
|  | Body mass | 1.674 | [-1.132; 4.556] | 0.218 |
|  | Geographic range size | -3.423 | [-5.789; -1.342] | <0.001 |
|  | Diet breadth | 0.013 | [-1.730; 1.723] | 0.968 |
|  | Habitat breadth | 0.208 | [-1.549; 1.990] | 0.782 |
|  | Litter size | -0.145 | [-2.739; 2.120] | 0.975 |
|  | Trophic level | -0.065 | [-2.059; 1.671] | 0.933 |
|  | Med. latitude (deg. 1)*^d^* | 0.307 | [-2.226; 2.799] | 0.813 |
|  | Med. latitude (deg. 2)*^d^* | -0.679 | [-2.918; 1.339] | 0.492 |
|  | Med. longitude (deg. 1)*^d^* | -0.323 | [-3.393; 2.508] | 0.849 |
|  | Med. longitude (deg. 2)*^d^* | -0.941 | [-3.369; 1.103] | 0.390 |
|  | Human pop. density*^e^* | 1.568 | [-0.057; 3.364] | 0.036 |
|  | Human pop. change*^f^* | 0.027 | [-1.591; 1.753] | 0.978 |
|  | Precipitation | 0.319 | [-1.282; 1.910] | 0.643 |

*^a^ See the Methods section and the database PanTHERIA, Jones et al. 2009, for details on each variable (all variables were scaled to a mean of 0 and a variance of 1); ^b^ in Million years of evolution; ^c^ number of known threat types affecting a species; ^d^ Median (Med.) latitudinal extent of each species range and median longitudinal extent of each species range, each expressed as orthogonal polynomials of degree (deg.) 1 and 2; ^e^ human population density; ^f^ mean rate of increase in human population density.*

Table S1-9. Ordinal phylogenetic model in a Bayesian MCMC framework testing the potential effects of 14 specified explanatory variables on species extinction risks with the following phylogenies: mamF= Fritz et al. phylogeny for mammals; F= pruned Fritz et al. phylogeny; R= pruned Rolland et al. phylogeny; AS= Springer et al. primate phylogeny with autocorrelated rates and soft-bounded constraints; IH= Springer et al. primate phylogeny with independent rates and hard-bounded constraints; IS= Springer et al. primate phylogeny with independent rates and soft-bounded constraints. Compared to Table S1-7, here we removed variables trophic level and diet breadth.

| *Phylogeny* | *Variable^a^* | *Posterior mean* | CI | pMCMC |
| --- | --- | --- | --- | --- |
| mamF | Distance to *H. sapiens^b^* | -0.236 | [-0.917; 0.462] | 0.488 |
|  | Originality | -0.033 | [-0.200; 0.203] | 0.728 |
|  | Nb of known threats*^c^* | 1.132 | [0.935; 1.294] | <<0.001 |
|  | Body mass | 0.797 | [0.538; 1.137] | <<0.001 |
|  | Geographic range size | -1.241 | [-1.521; -1.024] | <<0.001 |
|  | Habitat breadth | 0.092 | [-0.080; 0.253] | 0.248 |
|  | Litter size | -0.155 | [-0.427; 0.052] | 0.176 |
|  | Med. latitude (deg. 1)*^d^* | 0.053 | [-0.156; 0.224] | 0.616 |
|  | Med. latitude (deg. 2)*^d^* | -0.007 | [-0.189; 0.188] | 0.920 |
|  | Med. longitude (deg. 1)*^d^* | 0.226 | [0.026; 0.445] | 0.024 |
|  | Med. longitude (deg. 2)*^d^* | 0.005 | [-0.194; 0.177] | 0.968 |
|  | Human pop. density*^e^* | 0.039 | [-0.068; 0.165] | 0.504 |
|  | Human pop. change*^f^* | 0.047 | [-0.065; 0.177] | 0.520 |
|  | Precipitation | 0.057 | [-0.092; 0.229] | 0.528 |
| F | Distance to *H. sapiens^b^* | 0.140 | [-3.082; 3.200] | 0.913 |
|  | Originality | 0.341 | [-1.418; 2.259] | 0.659 |
|  | Nb of known threats*^c^* | 3.054 | [0.902; 5.307] | <<0.001 |
|  | Body mass | 1.499 | [-0.859; 3.835] | 0.189 |
|  | Geographic range size | -3.741 | [-6.041; -1.456] | <<0.001 |
|  | Habitat breadth | 0.745 | [-0.420; 2.028] | 0.183 |
|  | Litter size | 0.063 | [-1.930; 1.916] | 0.880 |
|  | Med. latitude (deg. 1)*^d^* | 0.094 | [-1.491; 1.855] | 0.958 |
|  | Med. latitude (deg. 2)*^d^* | -0.574 | [-1.921; 0.741] | 0.343 |
|  | Med. longitude (deg. 1)*^d^* | -0.554 | [-3.296; 1.790] | 0.646 |
|  | Med. longitude (deg. 2)*^d^* | 0.065 | [-1.762; 1.770] | 0.898 |
|  | Human pop. density*^e^* | 0.892 | [-0.183; 2.211] | 0.098 |
|  | Human pop. change*^f^* | 0.012 | [-1.183; 1.149] | 0.964 |
|  | Precipitation | -0.521 | [-1.747; 0.641] | 0.384 |
| R | Distance to *H. sapiens^b^* | 0.026 | [-2.530; 2.963] | 0.980 |
|  | Originality | 0.894 | [-0.388; 2.505] | 0.124 |
|  | Nb of known threats*^c^* | 1.992 | [0.714; 3.915] | <<0.001 |
|  | Body mass | 1.424 | [-0.474; 3.351] | 0.102 |
|  | Geographic range size | -2.980 | [-5.268; -1.191] | <<0.001 |
|  | Habitat breadth | 0.592 | [-0.270; 1.716] | 0.153 |
|  | Litter size | 0.428 | [-0.958; 1.901] | 0.413 |
|  | Med. latitude (deg. 1)*^d^* | -0.097 | [-1.266; 1.345] | 0.771 |
|  | Med. latitude (deg. 2)*^d^* | -0.404 | [-1.453; 0.595] | 0.367 |
|  | Med. longitude (deg. 1)*^d^* | -0.574 | [-2.753; 1.400] | 0.564 |
|  | Med. longitude (deg. 2)*^d^* | 0.291 | [-1.155; 1.660] | 0.589 |
|  | Human pop. density*^e^* | 0.610 | [-0.281; 1.719] | 0.150 |
|  | Human pop. change*^f^* | -0.069 | [-0.990; 0.742] | 0.898 |
|  | Precipitation | -0.439 | [-1.554; 0.466] | 0.402 |
| AS | Distance to *H. sapiens^b^* | 0.030 | [-2.948; 2.837] | 0.969 |
|  | Originality | 0.939 | [-0.417; 2.643] | 0.135 |
|  | Nb of known threats*^c^* | 2.148 | [0.520; 4.522] | <0.001 |
|  | Body mass | 1.445 | [-0.620; 3.521] | 0.135 |
|  | Geographic range size | -3.252 | [-5.505; -1.367] | <<0.001 |
|  | Habitat breadth | 0.578 | [-0.478; 1.833] | 0.232 |
|  | Litter size | -0.044 | [-2.015; 1.558] | 0.913 |
|  | Med. latitude (deg. 1)*^d^* | 0.503 | [-0.920; 2.325] | 0.522 |
|  | Med. latitude (deg. 2)*^d^* | -0.736 | [-2.084; 0.352] | 0.164 |
|  | Med. longitude (deg. 1)*^d^* | -0.159 | [-2.589; 1.953] | 0.958 |
|  | Med. longitude (deg. 2)*^d^* | -0.360 | [-2.003; 1.202] | 0.645 |
|  | Human pop. density*^e^* | 0.842 | [-0.284; 2.389] | 0.124 |
|  | Human pop. change*^f^* | -0.395 | [-1.747; 0.738] | 0.506 |
|  | Precipitation | -0.303 | [-1.511; 0.618] | 0.584 |
| IH | Distance to *H. sapiens^b^* | 0.071 | [-2.553; 2.770] | 0.917 |
|  | Originality | 0.706 | [-0.447; 2.109] | 0.158 |
|  | Nb of known threats*^c^* | 1.690 | [0.437; 3.791] | <<0.001 |
|  | Body mass | 1.380 | [-0.452; 3.133] | 0.094 |
|  | Geographic range size | -2.709 | [-4.618; -1.196] | <<0.001 |
|  | Habitat breadth | 0.399 | [-0.421; 1.359] | 0.286 |
|  | Litter size | 0.054 | [-1.609; 1.254] | 0.768 |
|  | Med. latitude (deg. 1)*^d^* | 0.374 | [-0.745; 1.886] | 0.573 |
|  | Med. latitude (deg. 2)*^d^* | -0.605 | [-1.758; 0.283] | 0.160 |
|  | Med. longitude (deg. 1)*^d^* | -0.015 | [-2.11; 1.763] | 0.912 |
|  | Med. longitude (deg. 2)*^d^* | -0.239 | [-1.643; 0.981] | 0.734 |
|  | Human pop. density*^e^* | 0.536 | [-0.301; 1.782] | 0.211 |
|  | Human pop. change*^f^* | -0.297 | [-1.404; 0.607] | 0.538 |
|  | Precipitation | -0.163 | [-1.059; 0.630] | 0.729 |
| IS | Distance to *H. sapiens^b^* | 0.129 | [-2.359; 2.728] | 0.886 |
|  | Originality | 0.679 | [-0.278; 2.014] | 0.137 |
|  | Nb of known threats*^c^* | 1.492 | [0.359; 3.310] | <0.001 |
|  | Body mass | 1.395 | [-0.084; 3.022] | 0.064 |
|  | Geographic range size | -2.546 | [-4.243; -1.095] | <<0.001 |
|  | Habitat breadth | 0.373 | [-0.335; 1.189] | 0.263 |
|  | Litter size | 0.124 | [-1.218; 1.270] | 0.682 |
|  | Med. latitude (deg. 1)*^d^* | 0.324 | [-0.739; 1.589] | 0.585 |
|  | Med. latitude (deg. 2)*^d^* | -0.550 | [-1.489; 0.327] | 0.150 |
|  | Med. longitude (deg. 1)*^d^* | 0.031 | [-1.813; 1.641] | 0.864 |
|  | Med. longitude (deg. 2)*^d^* | -0.197 | [-1.436; 0.904] | 0.763 |
|  | Human pop. density*^e^* | 0.444 | [-0.362; 1.458] | 0.257 |
|  | Human pop. change*^f^* | -0.256 | [-1.180; 0.549] | 0.546 |
|  | Precipitation | -0.128 | [-0.889; 0.589] | 0.762 |

*^a^ See the Methods section and the database PanTHERIA, Jones et al. 2009, for details on each variable (all variables were scaled to a mean of 0 and a variance of 1); ^b^ in Million years of evolution; ^c^ number of known threat types affecting a species; ^d^ Median (Med.) latitudinal extent of each species range and median longitudinal extent of each species range, each expressed as orthogonal polynomials of degree (deg.) 1 and 2; ^e^ human population density; ^f^ mean rate of increase in human population density.*

Table S1-10. Ordinal phylogenetic model in a Bayesian MCMC framework testing the potential effects of 14 specified explanatory variables on species extinction risks with the following phylogenies: mamF= Fritz et al. phylogeny for mammals; F= pruned Fritz et al. phylogeny; R= pruned Rolland et al. phylogeny; AS= Springer et al. primate phylogeny with autocorrelated rates and soft-bounded constraints; IH= Springer et al. primate phylogeny with independent rates and hard-bounded constraints; IS= Springer et al. primate phylogeny with independent rates and soft-bounded constraints. Compared to Table S1-9, here we removed threatened species not listed under criterion A of the IUCN Red List.

| *Phylogeny* | *Variable^a^* | *Posterior mean* | CI | pMCMC |
| --- | --- | --- | --- | --- |
| mamF | Distance to *H. sapiens^b^* | -0.289 | [-1.034; 0.498] | 0.432 |
|  | Originality | -0.106 | [-0.331; 0.111] | 0.336 |
|  | Nb of known threats*^c^* | 1.086 | [0.862; 1.317] | <<0.001 |
|  | Body mass | 1.085 | [0.666; 1.424] | <<0.001 |
|  | Geographic range size | -1.192 | [-1.528; -0.881] | <<0.001 |
|  | Habitat breadth | 0.219 | [0.020; 0.414] | 0.024 |
|  | Litter size | -0.144 | [-0.459; 0.160] | 0.336 |
|  | Med. latitude (deg. 1)*^d^* | 0.156 | [-0.072; 0.399] | 0.216 |
|  | Med. latitude (deg. 2)*^d^* | 0.014 | [-0.193; 0.278] | 0.880 |
|  | Med. longitude (deg. 1)*^d^* | 0.233 | [-0.039; 0.487] | 0.096 |
|  | Med. longitude (deg. 2)*^d^* | -0.121 | [-0.323; 0.116] | 0.224 |
|  | Human pop. density*^e^* | -0.061 | [-0.230; 0.121] | 0.496 |
|  | Human pop. change*^f^* | -0.137 | [-0.324; 0.053] | 0.128 |
|  | Precipitation | 0.232 | [0.002; 0.444] | 0.040 |
| F | Distance to *H. sapiens^b^* | 0.328 | [-2.969; 3.594] | 0.828 |
|  | Originality | 0.923 | [-1.129; 2.863] | 0.330 |
|  | Nb of known threats*^c^* | 4.137 | [1.914; 6.491] | <<0.001 |
|  | Body mass | 2.029 | [-0.373; 4.506] | 0.095 |
|  | Geographic range size | -3.451 | [-5.615; -1.488] | <<0.001 |
|  | Habitat breadth | 0.454 | [-0.861; 1.841] | 0.491 |
|  | Litter size | 0.158 | [-1.929; 2.258] | 0.849 |
|  | Med. latitude (deg. 1)*^d^* | -0.455 | [-2.613; 1.560] | 0.646 |
|  | Med. latitude (deg. 2)*^d^* | -0.904 | [-2.798; 0.791] | 0.305 |
|  | Med. longitude (deg. 1)*^d^* | -0.320 | [-3.002; 2.413] | 0.815 |
|  | Med. longitude (deg. 2)*^d^* | -0.468 | [-2.456; 1.293] | 0.637 |
|  | Human pop. density*^e^* | 1.865 | [0.173; 3.595] | 0.011 |
|  | Human pop. change*^f^* | -0.549 | [-2.148; 1.062] | 0.488 |
|  | Precipitation | 0.114 | [-1.216; 1.417] | 0.850 |
| R | Distance to *H. sapiens^b^* | 0.231 | [-2.784; 3.379] | 0.871 |
|  | Originality | 2.015 | [0.240; 3.978] | 0.023 |
|  | Nb of known threats*^c^* | 4.024 | [1.843; 6.294] | <<0.001 |
|  | Body mass | 1.919 | [-0.389; 4.227] | 0.093 |
|  | Geographic range size | -3.353 | [-5.353; -1.379] | <<0.001 |
|  | Habitat breadth | 0.412 | [-0.818; 1.718] | 0.516 |
|  | Litter size | 0.480 | [-1.245; 2.329] | 0.545 |
|  | Med. latitude (deg. 1)*^d^* | -0.255 | [-2.152; 1.695] | 0.776 |
|  | Med. latitude (deg. 2)*^d^* | -0.979 | [-2.757; 0.605] | 0.214 |
|  | Med. longitude (deg. 1)*^d^* | -0.453 | [-3.090; 2.139] | 0.714 |
|  | Med. longitude (deg. 2)*^d^* | -0.391 | [-2.237; 1.264] | 0.676 |
|  | Human pop. density*^e^* | 1.859 | [0.290; 3.630] | 0.008 |
|  | Human pop. change*^f^* | -0.572 | [-2.167; 0.771] | 0.429 |
|  | Precipitation | 0.213 | [-0.989; 1.441] | 0.707 |
| AS | Distance to *H. sapiens^b^* | 0.214 | [-2.725; 3.132] | 0.863 |
|  | Originality | 1.332 | [-0.259; 3.184] | 0.084 |
|  | Nb of known threats*^c^* | 3.143 | [1.005; 5.577] | <<0.001 |
|  | Body mass | 1.708 | [-0.504; 4.085] | 0.118 |
|  | Geographic range size | -3.209 | [-5.245; -1.168] | <<0.001 |
|  | Habitat breadth | 0.279 | [-1.036; 1.616] | 0.633 |
|  | Litter size | 0.281 | [-1.786; 2.178] | 0.695 |
|  | Med. latitude (deg. 1)*^d^* | 0.183 | [-1.686; 2.287] | 0.872 |
|  | Med. latitude (deg. 2)*^d^* | -0.669 | [-2.370; 0.908] | 0.368 |
|  | Med. longitude (deg. 1)*^d^* | -0.017 | [-2.582; 2.427] | 0.993 |
|  | Med. longitude (deg. 2)*^d^* | -0.828 | [-2.737; 0.920] | 0.351 |
|  | Human pop. density*^e^* | 1.422 | [0.138; 3.172] | 0.024 |
|  | Human pop. change*^f^* | -0.350 | [-1.814; 0.959] | 0.601 |
|  | Precipitation | 0.244 | [-1.031; 1.489] | 0.664 |
| IH | Distance to *H. sapiens^b^* | 0.153 | [-2.845; 3.026] | 0.896 |
|  | Originality | 1.148 | [-0.229; 2.877] | 0.089 |
|  | Nb of known threats*^c^* | 2.684 | [0.860; 4.962] | <<0.001 |
|  | Body mass | 1.623 | [-0.329; 3.692] | 0.090 |
|  | Geographic range size | -2.816 | [-4.753; -1.210] | <0.001 |
|  | Habitat breadth | 0.190 | [-0.862; 1.359] | 0.690 |
|  | Litter size | 0.296 | [-1.461; 1.883] | 0.625 |
|  | Med. latitude (deg. 1)*^d^* | 0.176 | [-1.507; 2.105] | 0.862 |
|  | Med. latitude (deg. 2)*^d^* | -0.621 | [-2.083; 0.744] | 0.332 |
|  | Med. longitude (deg. 1)*^d^* | 0.048 | [-2.382; 2.184] | 0.935 |
|  | Med. longitude (deg. 2)*^d^* | -0.633 | [-2.357; 0.846] | 0.415 |
|  | Human pop. density*^e^* | 1.088 | [-0.060; 2.464] | 0.042 |
|  | Human pop. change*^f^* | -0.313 | [-1.571; 0.858] | 0.589 |
|  | Precipitation | 0.250 | [-0.786; 1.323] | 0.615 |
| IS | Distance to *H. sapiens^b^* | 0.117 | [-2.790; 3.068] | 0.918 |
|  | Originality | 1.200 | [-0.310; 2.781] | 0.079 |
|  | Nb of known threats*^c^* | 2.609 | [0.856; 4.937] | <<0.001 |
|  | Body mass | 1.641 | [-0.331; 3.691] | 0.080 |
|  | Geographic range size | -2.799 | [-4.640; -1.207] | <0.001 |
|  | Habitat breadth | 0.205 | [-0.828; 1.288] | 0.661 |
|  | Litter size | 0.289 | [-1.407; 1.881] | 0.626 |
|  | Med. latitude (deg. 1)*^d^* | 0.215 | [-1.436; 2.067] | 0.835 |
|  | Med. latitude (deg. 2)*^d^* | -0.621 | [-2.026; 0.719] | 0.319 |
|  | Med. longitude (deg. 1)*^d^* | 0.042 | [-2.342; 2.155] | 0.929 |
|  | Med. longitude (deg. 2)*^d^* | -0.631 | [-2.295; 0.833] | 0.423 |
|  | Human pop. density*^e^* | 1.051 | [-0.073; 2.334] | 0.042 |
|  | Human pop. change*^f^* | -0.287 | [-1.571; 0.826] | 0.617 |
|  | Precipitation | 0.247 | [-0.853; 1.279] | 0.627 |

*^a^ See the Methods section and the database PanTHERIA, Jones et al. 2009, for details on each variable (all variables were scaled to a mean of 0 and a variance of 1); ^b^ in Million years of evolution; ^c^ number of known threat types affecting a species; ^d^ Median (Med.) latitudinal extent of each species range and median longitudinal extent of each species range, each expressed as orthogonal polynomials of degree (deg.) 1 and 2; ^e^ human population density; ^f^ mean rate of increase in human population density.*
